# Induced variation in *BRASSINOSTEROID INSENSITIVE 1* (*BRI1*) confers a compact wheat architecture

**DOI:** 10.1186/s12870-025-06762-w

**Published:** 2025-05-26

**Authors:** Manpartik S. Gill, Andrew L. Phillips, Danuše Tarkowská, John Addy, Patrycja Sokolowska, M. John Foulkes, Stephen Pearce, Stephen G. Thomas, Peter Hedden

**Affiliations:** 1 Sustainable Soils & Crops, Rothamsted Research, Harpenden, AL5 2JQ UK; 2 Laboratory of Growth Regulators, Palacký University Olomouc & Institute of Experimental Botany, Czech Academy of Sciences, Šlechtitelů 27, Olomouc, 779 00 Czech Republic; 3 School of Biosciences, Division of Plant and Crop Sciences, University of Nottingham, Sutton Bonington, LE12 5RD UK

**Keywords:** Wheat, Brassinosteroids, EMS-mutagenesis, BR insensitivity, Upright leaf angles, Semi-dwarf

## Abstract

**Background:**

The brassinosteroid (BR) plant hormones regulate numerous developmental processes, including those determining stem height, leaf angle, and grain size that have agronomic relevance in cereals. Indeed, barley (*Hordeum vulgare*) varieties containing *uzu* alleles that impair BR perception through mutations in the BR receptor BRASSINOSTEROID INSENSITIVE 1 (BRI1) exhibit a semi-dwarf growth habit and more upright leaves suitable for high-density planting. We used forward and reverse genetic approaches to develop novel *BRI1* alleles in wheat (*Triticum aestivum* L.) and investigated their potential for crop productivity improvement.

**Results:**

The combination of ethyl methanesulfonate-induced mutations introducing premature stop codons in all three homoeologous *TaBRI1* genes resulted in severe dwarfism, malformed leaves and sterility as observed in *bri1* mutants in other species. Double mutants had reduced flag-leaf angles (FLAs) conferring a more upright canopy but exhibited no differences in height or grain weight. In a targeted forward genetics screen using a double mutant, we identified two BR-insensitive lines with reduced height and FLA that contained amino acid substitutions in conserved regions of BRI-A1. The less severe mutant had a 56% reduction in FLA and was 35% shorter than the wild type, although seed set, seed area and grain weights were also reduced. The most severe mutants contained elevated levels of bioactive BRs and increased expression of BR-biosynthesis genes consistent with reduced feedback suppression of biosynthesis.

**Conclusion:**

Our study gives a better understanding of BRI1 function in wheat and provides mutants that could potentially be explored for improving grain yields when sown at high density.

**Supplementary Information:**

The online version contains supplementary material available at 10.1186/s12870-025-06762-w.

## Background

Brassinosteroids (BRs) are a class of plant steroids, which includes bioactive compounds such as brassinolide (BL) and castasterone (CS) that function as hormones with essential roles in plant growth and development [1]. Other BRs are intermediates in the biosynthesis pathways or inactivated products of BR catabolism. In cereals, BRs regulate many important agronomic traits such as stem elongation, leaf angle, grain size, flowering time, and senescence [2, 3].

Bioactive BRs are perceived by the transmembrane receptor BRASSINOSTEROID INSENSITIVE1 (BRI1), a leucine-rich repeat (LRR) serine-threonine (Ser/Thr) protein kinase [4]. In Arabidopsis (*Arabidopsis thaliana*), BRI1 contains three domains that are essential for its function: extracellular, transmembrane, and Ser/Thr-kinase [5]. Biologically active BRs bind to the BRI1 extracellular domain forming a groove (containing an island domain spanning 70 amino acids and five LRRs) which induces autophosphorylation. Phosphorylated BRI1 then associates with its co-receptor BRI1-ASSOCIATED KINASE 1 (BAK1) and disassociates from the negative regulator BRI1 KINASE INHIBITOR 1 (BKI1) [6]. Transphosphorylation between BRI1 and BAK1 initiates a signalling cascade that maintains the transcription factors BRASSINAZOLE-RESISTANT 1 (BZR1) and BR-INSENSITIVE-EMS-SUPPRESSOR 1/BRASSINAZOLE-RESISTANT 2 (BES1/BZR2) in a dephosphorylated state in which they are sequestered in the nucleus and bind to the promoters of target genes [6]. The BR biosynthetic pathway is regulated by bioactive BRs through a homeostatic feedback mechanism. External BR application leads to the downregulation in the expression of sterol and BR biosynthetic genes, including *HYDRA2 (HYD2)*, *DWARF7 (DWF7)*, *DWF4*, *DWF5*, *CONSTITUTIVE PHOTOMORPHOGENESIS AND DWARFISM* (*CPD*), *EBISU DWARF* (*D2)* and *BR6OX1,2* [7–10]. This homeostatic mechanism is regulated via BZR1 and BZR2, which bind to the promoters of BR biosynthesis genes to suppress their expression [11–13].

The *BRI1* genes in hexaploid bread wheat (*Triticum aestivum* L.) encode proteins that have high homology with orthologues in the monocot species barley (*Hordeum vulgare*) (95% amino acid identity) and rice (*Oryza sativa*) (83%), and with Arabidopsis (54%), which differs from the monocot BRI1 proteins in having 25 tandem LRRs in the extracellular domain rather than 22 in monocots [14].

In cereals, some mutant *bri1* alleles confer beneficial traits and have been exploited in breeding. Severe mutant alleles of *OsBRI1* such as *d61-3*, which encodes a protein with an amino acid substitution, H420P, within the LRR region, and *d61-4*, which encodes a truncated protein due to the introduction of a premature stop codon, E847*, within the kinase domain, result in extreme dwarfism, sterility and malformed leaves [15]. However, a weaker allele, *d61-7* (A467V in the LRR region) confers more upright leaves and a semi-dwarf stature, yielding 35% higher biomass compared to wild-type when grown at high planting density. However, the *d61-7* mutant also has smaller grains so does not lead to overall higher grain yields [16] By contrast, *uzu* barley landraces, which carry mutations in *HvBRI1*, have been in cultivation for over a century in central and southern Japan and southern coastal parts of Korea [17, 18]. The *uzu1.a* allele which carries an H857R substitution in the kinase domain [19] confers a 20% height reduction, due to restricted internode elongation, and a more upright canopy architecture, thus supporting dense planting and heavy manuring, leading to higher biomass and lodging resistance under field conditions [17]. Due to these favourable traits, by the 1930s *uzu* varieties were grown in > 70% of arable land under barley cultivation in Japan and > 30% in the Korean peninsula [20]. By the early 2000s, all the cultivated hull-less barley varieties grown in southern Japan were of the *uzu* type. In China, 68.4% of semi-dwarf barley varieties released since 1950 carry *uzu* alleles [21]. Thus, altering BRI1 activity has considerable potential for tailoring plant architecture for more favourable distribution of light among canopy leaf layers for photosynthesis and/or enhanced partitioning of assimilate to the ear through restricted internode elongation and ultimately crop improvement but to date has not been exploited in wheat breeding programmes.

The homoeologous *TaBRI1* wheat genes are located on the long arms of chromosomes 3A, 3B and 3D [14]. Plants carrying deletions in *TaBRI1-A1* or *TaBRI1-D1*, produced by ion beam mutagenesis, exhibit erect leaf architecture during seedling development and across the reproductive stages (0 to 30 days post anthesis) as well as a significant reduction in final plant height, thousand-grain weight (TGW) and harvest index [22]. The numbers of spikelets and grains per spike were unaffected. Additionally, these mutants had reduced photosynthetic efficiency and increased susceptibility to high light and temperature stresses [22]. However, ion-beam mutagenesis induces large deletions in the genome which can confound mutant characterisation. Thus, there is a need for a full range of backcrossed, stable single, double, and triple *tabri1* mutants to explore the potential of manipulating *TaBRI1* for wheat genetic improvement.

In the current study, we used reverse genetics to identify loss-of-function mutations in all three homoeologous *TaBRI1* genes and found that different combinatorial mutants confer a more erect growth habit in both glasshouse and field conditions. We also identified two novel alleles with point mutations in the LRR and Ser/Thr-kinase domains of *TaBRI1-A1* that, in combination with loss-of-function *TaBRI1-B1* and *TaBRI1-D1* alleles, confer reduced BR sensitivity, erect growth and semi-dwarf phenotypes. This study characterises the role of *BRI1* in regulating wheat growth and development and highlights the potential for introducing beneficial traits in this species through manipulating BR signalling.

## Materials and methods

### Plant materials and growth conditions

#### Growth conditions under glasshouse experiment

The spring wheat cultivar Cadenza, supplied by CPB Twyford Ltd, was used in all experiments. For the GH2021, GH2022 and lamina joint inclination assays, wheat seeds were imbibed on damp filter paper at 4 ℃ for 4–5 d then transferred to soil in 15 cm pots (75% peat, 12% sterilised loam, 3% vermiculite and 10% grit) and grown in GH conditions (18–20 ℃ day/14–15 ℃ night temperature, 16 h photoperiod).

For BR quantification and RNA-seq experiments, germinated seeds were transferred to damp vermiculite in a controlled environment (CE) chamber (Constant 21 ℃ and 24 h photoperiod using fluorescent light source set at 300 μmolm^−2^ s^−1^ PAR). The entire above-ground tissue was harvested from seedlings at the 2nd leaf stage in liquid nitrogen.

### Screening Cadenza TILLING population

Premature stop codon mutations in *TaBRI1-A1*, *TaBRI1-B1* and *TaBRI1-D1* genes were identified in a mutagenized Cadenza population from an online database (http://plants.ensembl.org/Triticum_aestivum/). DNA from M2 lines, produced at Rothamsted Research, was subjected to exon capture and sequencing to identify mutations [23]. M3 seed was supplied to John Innes Institute who generated and supplied the M4 seed, which was inter-crossed to combine mutations. F_1_ plants (AaBbDd) were backcrossed 3 × to non-mutagenized Cadenza to reduce background mutations then selfed to obtain a BC_3_F_2_ population segregating for various combinatorial mutants (*tabri1-a.1*, *tabri1-b* and *tabri1-d*, *tabri1-a.1b*, *tabri1-a.1d* and *tabri1-bd*, *tabri1-a.1bd* and *TaBRI1-NS*).

### Targeted forward genetics screen

Seeds of the *tabri1-bd* double knockout mutant were treated with 0.4% EMS as described previously [24, 25]. The M_1_ seed was planted in the field in March 2018 with a sowing density of 100 grains/m^2^ in twelve separate plots (1.8 × 12 m plots). At maturity, the M_2_ seed was harvested from each plot to generate twelve individual M_2_ bulk pools which were sown in the field in March 2019. Eight plots of 1.8 × 12 m per pool were sown at a planting density of 100 grains /m^2^. Visual screening of the plots was conducted from GS65 to GS87 to identify mutants displaying altered height and/or leaf erectness in individual M_2_ plants. M_3_ seed was collected from selected M_2_ plants at maturity which were then grown in GH conditions at M_3_ and M_4_ stage.

### Phenotypic characterisation of *tabri1* mutants under glasshouse conditions

*Tabri1* mutants were phenotyped in GH conditions using 6–8 biological replicates which were sown in a randomised complete block design. Final plant height was recorded as the length of the tallest tiller from the base of the stem touching the soil to the tip of the spike at maturity. Internode and spike length (from the base of the first spikelet to the top of the terminal spikelet just beneath the base of the awns) was taken from 2–3 tallest tillers per plant. The number of spikelets producing grain per spike was also measured. Spikelet density was calculated by dividing the number of spikelets per spike by the spike length. The leaf angle was defined as the angle between the vertical stem and the midrib of the leaf measured at the seedling stage (2nd leaf) and reproductive stage on the flag leaf. For measuring flag leaf angles (FLA) the date of ear emergence and anthesis was recorded on a representative primary tiller. The leaf angle was measured using a protractor and was recorded on the primary tiller at ear emergence (GS-55), anthesis (GS-61), completion of anthesis (GS-69), soft dough (GS-77), late milk (GS-85) and ripening stage (GS-93). The Marvin grain analyser (INDOSAW, India) was used to determine grain area using 3–5 g of clean threshed seed. TGW was measured by weighing 1,000 clean-threshed grains.

### Field assessment of wheat *tabri1* mutants

The genotypes *tabri1-a.1, tabri1-b, tabri1-d, tabri1-a.1b, tabri1-a.1d, tabri1-bd, tabri1-a.3bd* and *TaBRI1-NS* were sown on 15th April 2022 at Rothamsted Research in 1 m^2^ plots as a randomised complete block design with four replicates. All experiments were grown with standard farm practice for fertiliser and pesticides but with no plant growth regulators applied. Final plant height, spike length, number of spikelets/spike, grain area and grain weight were measured as in the GH experiment using 10–15 plants per plot. FLA was measured in 10–15 uniform tillers using a protractor. The final plant height was recorded on the 10 tallest tillers per plot.

### Brassinosteroid hormone profiling

Bioactive and precursor BR concentrations were measured in 2nd leaf seedling tissues of *tabri1-a1.bd*, *tabri1-a.2bd*, *tabri1-a.3bd, tabri1-bd* and *TaBRI1-NS*. Multiple plants were pooled to obtain sufficient tissue for five biological replicates. Leaf tissue was weighed frozen in liquid N_2_ and freeze-dried. BRs were extracted, purified, and analysed by UPLC-ESI–MS/MS as described in [26].

### Genotyping

Genomic DNA was extracted from seedling tissue using a CTAB-buffer extraction.

*TaBRI1* genes were amplified using 0.05 μM of homoeologue-specific PCR primers (Table S2 and S3) with Hot Shot Diamond Mastermix (Clent Life Science, Stourbridge, UK) and annealing temperature between 55–65 ℃ depending on the primer pair (Table S2 and S3).

PCR products were purified using QIAquick PCR Purification kit (Qiagen, Hilden, Germany). and sequenced by Eurofins Genomics (Wolverhampton, U.K.).

We selected *bri1* mutations using KASP assays (Table S4). Each assay was run using KASP low-ROX Mastermix (LGC, Teddington, UK) with primers specific to each SNP with FAM, HEX or VIC tails and a common reverse primer with 37 cycles of touchdown PCR. Plates were read using the 7500 Fast Software v2.3 (Applied Biosystems, Foster City, California, USA) and analysed using the KlusterCaller™ software (LGC, Teddington, UK).

### RNA sequencing

The same tissues used for BR profiling were used to extract RNA using a Monarch® Total RNA Miniprep Kit (New England Biolabs, Ipswich, Massachusetts, USA) according to the manufacturer’s instructions and including DNase treatment. To assess RNA concentration and quality, Agilent 2100 Bioanalyzer (Agilent, Santa Clara, California, USA) and Agilent 6000 Nano RNA Kit (Agilent, Santa Clara, California, USA) was used according to the manufacturer’s instructions. RNA-seq was performed by Novogene Bioinformatics Technology Co. Ltd. (Cambridge, UK). Paired reads were trimmed to remove adaptor sequences and for quality using Trimmomatic 0.39 software [27] (SLIDINGWINDOW:4:20; MINLEN:50). The raw FASTQ files were mapped to wheat IWGSC RefSeq v2.1 genome assembly using STAR 2.7.8a [28] (outFilterMismatchNmax 6; – alignIntronMax 10000). Raw mapped reads were counted using HTSeq 0.11.3 [29] and converted to TPMs (transcripts per million). Differential expression between each mutant genotype and *TaBRI1-NS* was performed using DESeq2 [30] in RStudio version 4.1.1717 (RStudioTeam, 2015). Genes with > two-fold change in expression and FDR-adjusted *P*-value of ≤ 0.05 were classified as DEGs. PCA plot were generated using Bioconductor package pheatmap version 1.0.12 (Kolde, R. *Pheatmap: pretty heatmaps*. R Package Version 1.0.12. https://CRAN.R-project.org/package=pheatmap (2012). Differential expression was visualized by generating heatmaps for the BR-pathway genes as reported by [31, 32] using Heatmapper [33], http://www.heatmapper.ca/). Venny2.1 [34], https://csbg.cnb.csic.es/BioinfoGP/venny.html) was used to produce Venn diagrams for the genes co-expressed in the *tabri1* mutants compared to *TaBRI1-NS*. g:Profiler (http://biit.cs.ut.ee/gprofiler/, [35] was used to visualize gene ontology (GO) terms.

### Lamina joint inclination assay

Two-cm sections containing the 2nd leaf lamina joint, leaf sheath, and leaf blade were excised from uniformly developed seedlings and floated in petri dishes containing autoclaved water for 10 min. The samples were transferred to 10^–5^ M 24-*epi*brassinolide (*epi*BL) solution or autoclaved water in Petri dishes, sealed using parafilm and transferred to an incubator set at 29 °C in the dark for 2 d. The leaf angles were recorded using a protractor [36].

### Statistical analysis

The mutants along with wild-type *TaBRI1-NS* were planted in randomised and replicated blocks to reduce statistical error in GH and field experiments. Analysis of variance was conducted for estimating statistical differences between genotypes and treatments. ANOVA was performed using Genstat software (21.1st Edition) to determine *P*-values, SED, and LSD at a 5% level of significance. The field data were analysed using unbalanced ANOVA using regression (Genstat software 21.1st Edition). Statistically significant differences between mutants and controls were further tested from Fisher’s LSD unprotected test providing *P*-values. Paired *t*-tests were performed using Graphpad Prism software (version 9.4.0) to test means before and after treatment which yielded *P*-values. Mean and residual plots for each dataset were generated to access the normality. The graphs using individual values or means and *P*-values (obtained using Fisher’s unprotected LSD test) were made in Graphpad Prism software (version 9.4.0).

## Results

### Generation of *tabri1* mutants

**Fig. 1:**
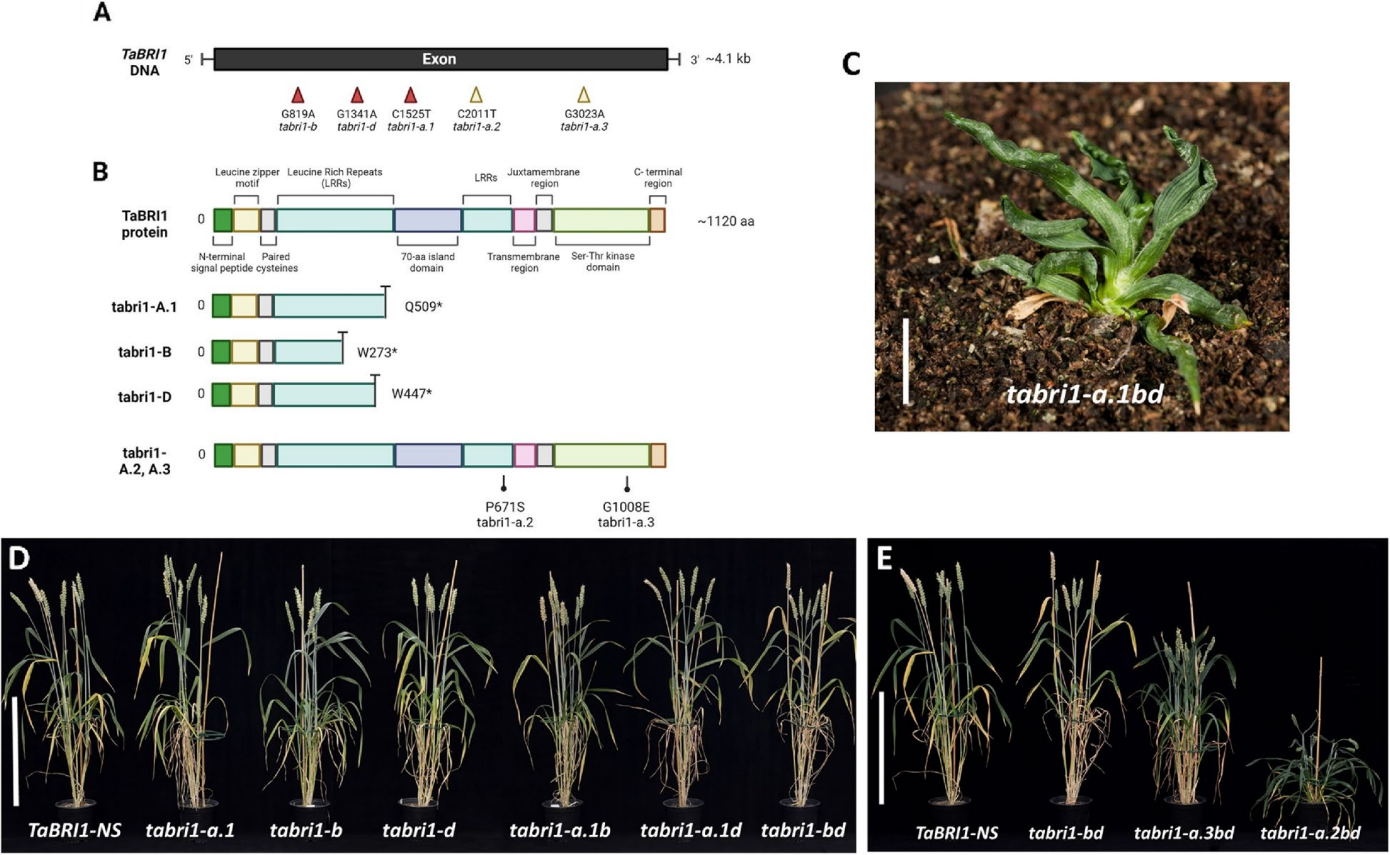
*tabri1* mutants developed via reverse- and forward-genetics approaches (**A**) *TaBRI1* gene indicating the position of nonsense mutations in the three homoeologues (red arrows) and missense mutations identified in *TaBRI1-A1* (yellow arrows) (**B**) Structure of the TaBRI1 protein showing conserved domains and the predicted TaBRI1 proteins encoded by the mutant alleles. The positions of the amino acid substitutions in *tabri1-a.2bd* (P671S) and *tabri1-a.3bd* (G1008E) are indicated. Produced using Biorender.com. **C** Phenotype of the triple *tabri1-a.1bd* mutant at two months after germination growing under glasshouse conditions (bar length is 2.5 cm). The phenotype of (**D**) the segregating wild-type (*TaBRI1-NS*) with single and double mutants (**E**) the triple mutants (*tabri1-a.2bd* and *tabri1-a.3bd*) and a representative double mutant (*tabri1-bd*). Plants were photographed at GS83. Bar length is 40 cm

We developed a *tabri1* loss-of-function mutant in hexaploid wheat by combining three lines carrying mutations, derived from the Cadenza EMS population [23], that introduce premature stop codons in the coding sequence of *TaBRI1-A1*, *TaBRI1-B1*, and *TaBRI1-D1* (Fig. 1A, Table 1). All three mutant alleles encode truncated proteins lacking the transmembrane and Ser/Thr-kinase domains which are essential for protein function (Fig. 1B). The *TaBRI1* mutations were combined by inter-crossing and backcrossed three times to generate a BC_3_F_2_ population segregating for each mutation. We selected all single (*tabri1-a.1*, *tabri1-b,* and *tabri1-d*), double (*tabri1-a.1b*, *tabri1-a.1d* and *tabri1-bd*) and triple (*tabri1-a.1bd*) mutant combinations, as well as the segregating wild-type null segregant line (*TaBRI1-NS*) from this population for phenotypic characterisation.

**Table 1:** *BRI1* homoeologues and mutant alleles used in this study

Gene	Gene ID	Mutant name	Amino acid substitution	Cadenza TILLING Line
*TaBRI1-A1*	TraesCS3A02G245000	*tabri1-a.1*	Q509^a^	0802
		*tabri1-a.2*	P671S (0.00)	-
		*tabri1-a.3*	G1008E (0.00)	-
*TaBRI1-B1*	TraesCS3B02G275000	*tabri1-b*	W273^a^	0313
*TaBRI1-D1*	TraesCS3D02G246500	*tabri1-d*	W447^a^	0119

The position of nonsense mutations identified from the Cadenza TILLING population and missense mutations identified from our targeted forward genetics screen are indicated. Sorting Intolerant From Tolerant (SIFT) scores for amino acid substitutions are shown in parentheses, where values less than 0.05 indicate that the mutation is predicted to have a deleterious effect on protein function

^a^stop codon

Figure 1 shows the gross morphology of single, double, and triple mutants along with the wild-type *TaBRI1-NS* at the grain-filling stage growing under glasshouse conditions. The *tabri1-a.1bd* triple mutant was infertile and displayed a severe dwarf phenotype with malformed leaves, demonstrating that the wheat *BRI1* genes are essential for normal development (Fig. 1C). The height of the single and double mutants was comparable to that of *TaBRI1-NS* (with some small, non-replicable differences as discussed below), demonstrating a high level of functional redundancy for plant height amongst the *TaBRI1* homoeologues (Fig. 1D).

### Identification of novel *TaBRI1-A1* mutants from a targeted forward genetics screen

The high level of functional redundancy among the *TaBRI1* homoeologues and the severe dwarf phenotype of the triple loss-of-function mutant prompted us to identify weaker mutations that might confer a potentially useful ideotype combining a semi-dwarf growth habit with upright leaves. We performed a targeted forward genetics screen by mutagenizing the *tabri1-bd* double mutant and identified two lines, *M3-31* and *M3-49*, which were shorter than both *TaBRI1-NS* and *tabri1-bd* and had more upright leaves (Fig. 1E). *M3-49* displayed the milder mutant phenotype (Fig. 1E) while in *M3-31* the leaves were dark green and twisted (Fig. 1E and Additional file 1: Figure S1) which is characteristic of BR signalling mutants in other cereals [16, 37, 38]. Unlike the *tabri1-a.1bd* mutant, both lines transitioned to the reproductive stage and set seeds. We hypothesized that these phenotypes might be due to mutations in *TaBRI1-A1* that reduce function. Sequencing of the *TaBRI1-A1* open reading frame revealed that *M3-31* carries a G to A point mutation resulting in a P671S substitution in the 22nd LRR and that *M3-49* carries a C to T point mutation resulting in a G1008E substitution in the Ser/Thr-kinase domain (Fig. 1B). The mutant alleles were named *tabri1-a.2bd* (*M3-31*) and *tabri1-a.3bd* (*M3-49*) (Table 1). We crossed both mutants to the *tabri1-bd* mutant and selected triple mutants from the BC_1_F_2_ generation for phenotypic characterisation.

### Phenotypic characterization of *tabri1* mutants under glasshouse conditions

We phenotyped the wheat *bri1* mutants in glasshouse conditions (GH) during 2021 and 2022. The data collected from GH2021 are presented below and data from GH2022 are presented in Additional file 1: Figure S2. We observed some differences in the traits such as final plant height and TGW between GH2021 and GH2022, possibly due to warmer and brighter conditions during GH2022 (Sheet 1 in Additional File S2). Despite these differences, the genotypic effects were consistent between years.

**Fig. 2:**
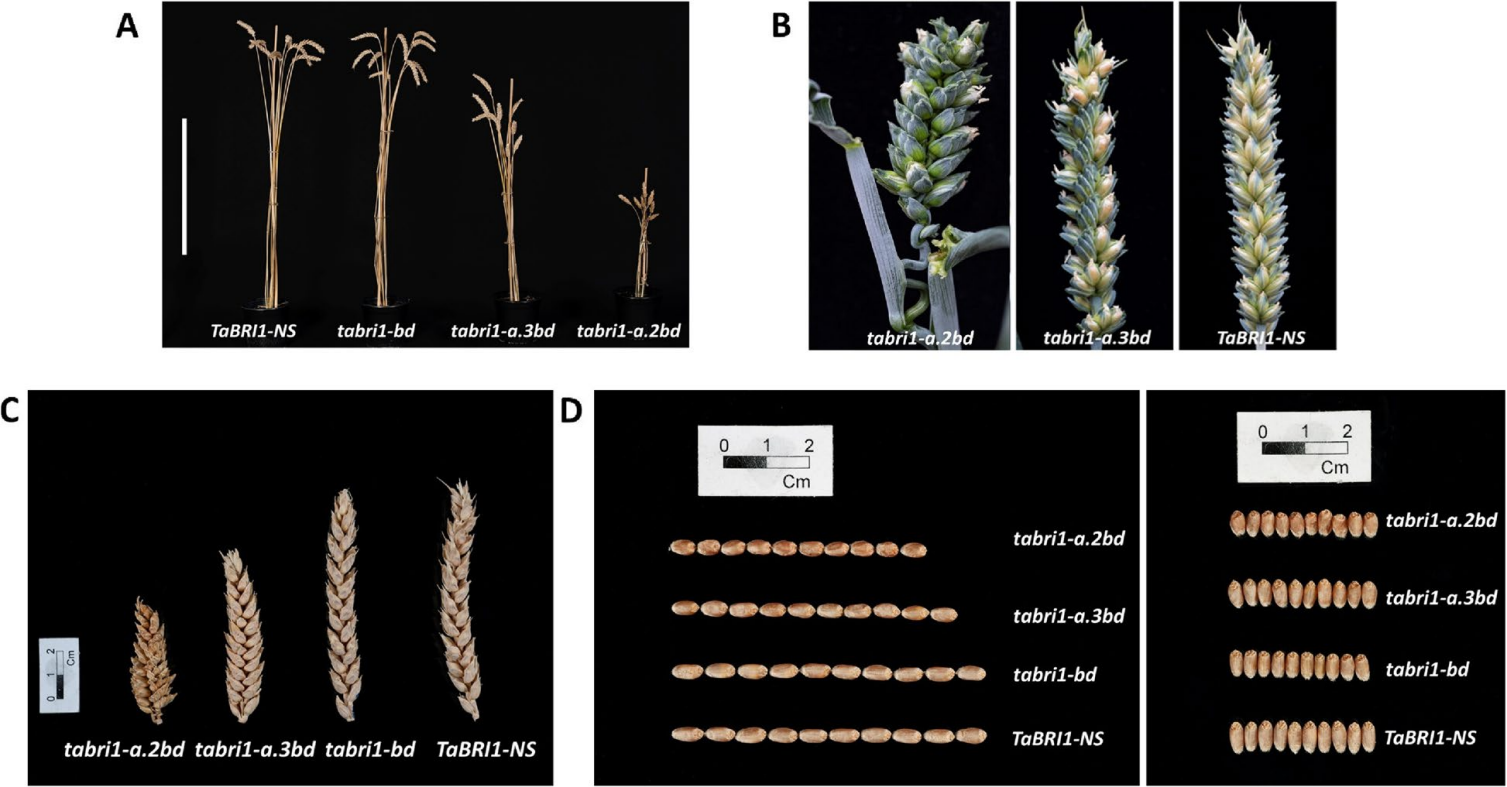
Phenotype of novel *tabri1* triple mutants. **A** Mature stems of the triple mutants (*tabri1-a.2bd* and *tabri1-a.3bd*), a representative double mutant (*tabri1-bd*) and control *TaBRI1-NS* from GH2021 photographed at GS93. Bar length is 40 cm.** B** Spikes from *tabri1-a.2bd* and *tabri1-a.3bd* compared to *TaBRI1-NS* at GS83 (soft dough stage) (**C**) Spikes and (**D**) grains (10 grains photographed end-to-end and side-by-side) from the triple mutants (*tabri1-a.2bd* and *tabri1-a.3bd*), representative double mutant (*tabri1-bd*) and control *TaBRI1-NS* photographed at maturity

**Fig. 3:**
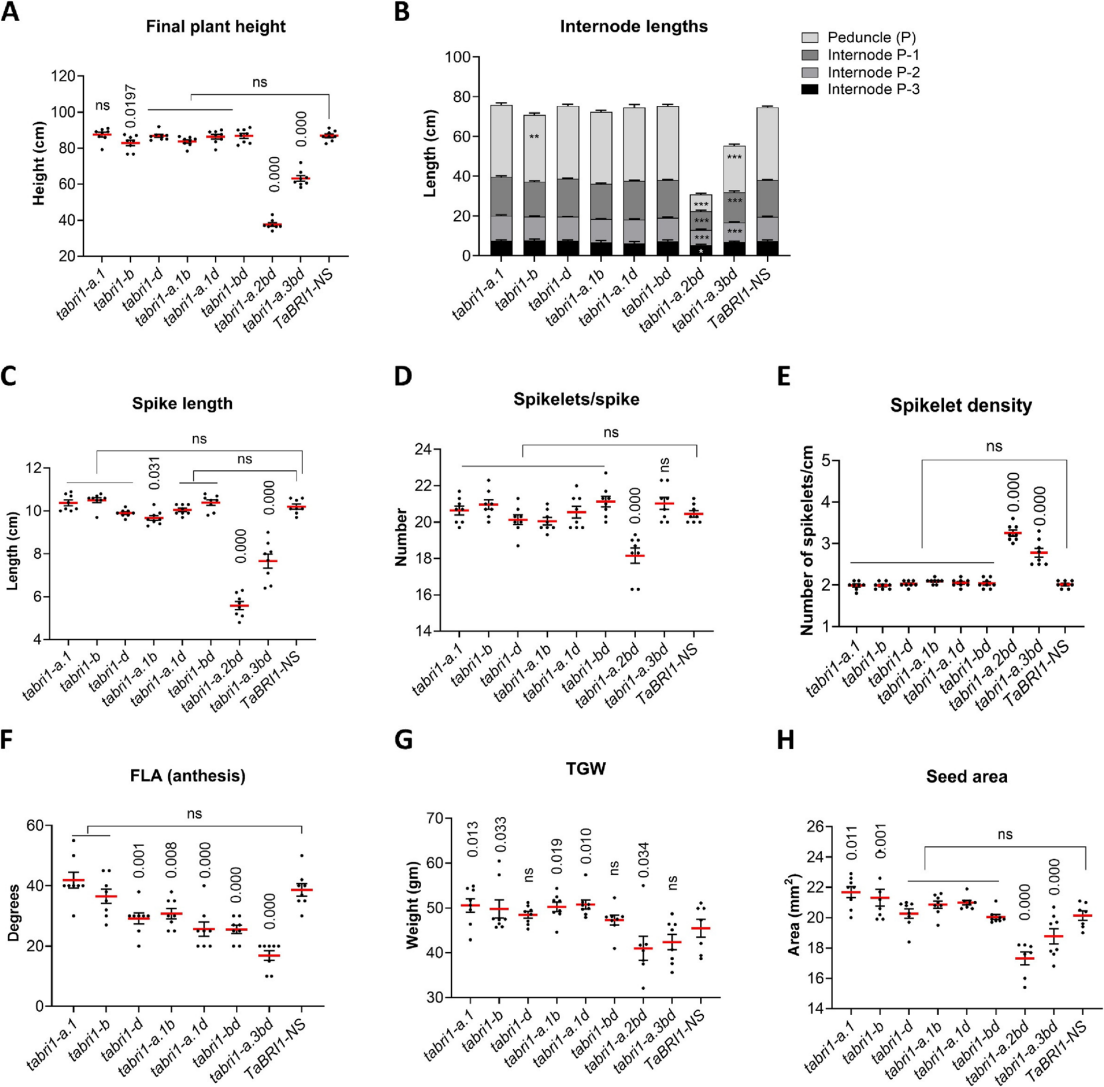
Phenotypic characterisation of *tabri1* mutants in GH2021. **A** Final plant height at maturity (*n* = 8). **B** Internode lengths recorded at maturity (*n* = 8). **C** Spike length at maturity (*n* = 8). **D** Number of spikelets per spike (*n* = 8). **E** Spikelet density (*n* = 8). **F** FLA at anthesis (*n* = 8). **G** TGW in mature grains (*n* = 8). **H** Area of mature grains (*n* = 8). ***, ** and * indicate significant difference from *TaBRI1-NS*, *P* < 0.001, 0.01 > *P* > 0.001 and 0.05 > *P* > 0.01, respectively. *P*-values were obtained using Fisher’s unprotected LSD test

The *tabri1-a.2bd* and *tabri1-a.3bd* mutants were 57% and 27% shorter, respectively, than *TaBRI1-NS* (Figs. 2A and 3A). This height difference was caused by significant reductions in the lengths of the spike, peduncle (P), and upper internodes in both mutants (Figs. 2C, 3B and C). The number of spikelets per spike was reduced in *tabri1-a.2bd*, but only in GH2021 (Fig. 3D) and increased in *tabri1-a.1b* and *tabri1-a.1d*, only in GH2022 (Additional file 1: Figure S2) compared to *TaBRI1-NS*. The reductions in spike length in these mutants with a small reduction in spikelet number in *tabri1-a.2bd*, but not in *tabri1-a.3bd*, resulted in increased spikelet densities (Fig. 3E). Otherwise, spike morphology was not affected. By contrast, final plant height was not significantly different in any single or double *tabri1* mutant compared to *TaBRI1-NS* in either experimental replication, except for *tabri1-b* and *tabri1-a.1d* mutants which were significantly shorter than *TaBRI1-NS* in GH2021 and GH2022, respectively (Fig. 3A and Additional file 1: Figure S2). Spike length was unaffected in the single and double mutants, except for *tabri1-a.1b* and *tabri1-b* mutants in which spikes were significantly shorter than *TaBRI1-NS* in GH2021 and GH2022, respectively (Fig. 3C, Additional file 1: Figure S2). This result indicates *BRI1* genes are functionally redundant in controlling stem and rachis elongation in wheat.

All double *tabri1* mutants exhibited significantly reduced flag leaf angles (FLA) conferring a more upright architecture compared to *TaBRI1-NS* (Fig. 3F). The effect was strongest in the *tabri1-a.3bd* triple mutant where FLA was reduced by 56% (Fig. 3F). Among single *bri1* mutants, only *bri1-d* exhibited significantly reduced FLA and only in the GH2021 experiment (Fig. 3F). It was not possible to record the leaf angle in the *tabri1-a.2bd* triple mutant due to its twisted and disoriented leaves (Additional file 1: Figure S1). Relative differences in FLA were maintained in two experimental replications (Additional file 1: Figure S2) and were consistent at ear emergence, anthesis, watery endosperm, soft dough, and ripening stages (Additional file 1: Figure S3).

While TGW was significantly higher in all three single mutants and the *tabri1-a1.d* double mutant, the *tabri1-a.2bd* and *tabri1-a.3bd* triple mutants exhibited significant reductions compared to *TaBRI1-NS* (Fig. 3G and Additional file 1: Figure S2). These results were consistent with changes in seed area (Figs. 2D, 3H and Additional file 1: Figure S2).

### Phenotypic characterization of *tabri1* mutants under field conditions

**Fig. 4:**
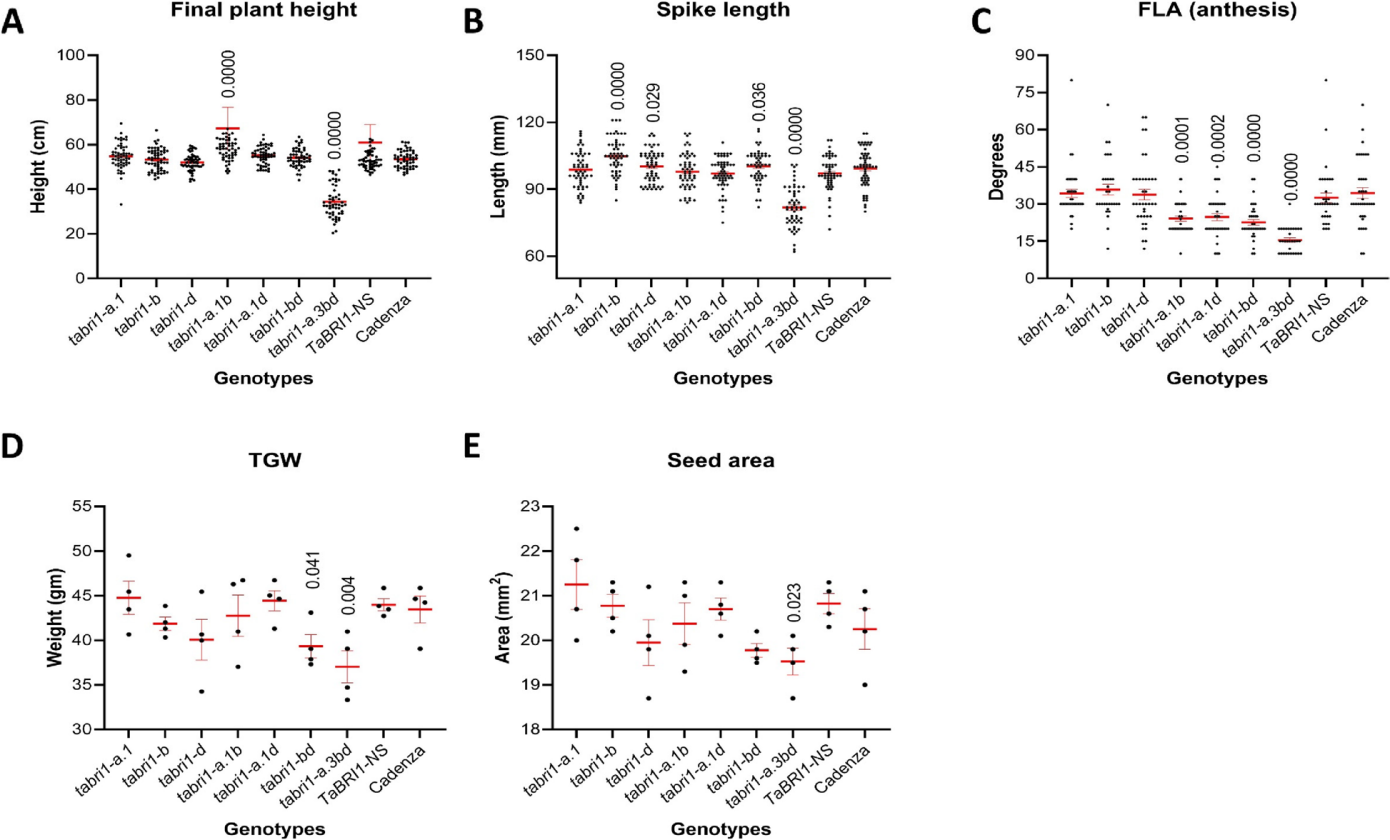
Phenotypic characterisation of *tabri1* mutants in a field trial in 2022**.** A Final plant height at maturity (*n* = 55–60). **B** Spike length at maturity (*n* = 55–60). **C** FLA at anthesis (*n* = 40). **D** TGW of mature grains (*n* = 4). **E** Seed area of mature grains (*n* = 4). *P*-values for significant differences between mutants and *TaBRI1-NS*, were obtained using Fisher’s unprotected LSD test

The phenotypes of some selected *tabri1* mutants were assessed under field conditions in 2022. The triple mutant *tabri1-a.3bd* was significantly shorter than *TaBRI1-NS* (34.3 cm compared with 53.0 cm, a 35% reduction) (Fig. 4A). There were no significant differences in final height between the controls and the single and double mutants, except for *tabri1-a.1b* which was 8.5% taller than *TaBRI1-NS* (although this was not observed in the glasshouse experiments). Spikes were 16% shorter in the *tabri1-a.3bd* compared to *TaBRI1-NS* (Fig. 4B). Significant increases in spike length compared with *TaBRI1-NS* were found in *tabri1-bd* (5%), *tabri1-d* (4.7%), and *tabri1-b* (9.4%), although apart from *tabri1-b*, for which a longer spike was also noted in GH2022, these differences were not observed in the glasshouse experiments.

All *tabri1* double and triple mutants exhibited significant reductions in FLA compared to the *TaBRI1-NS* conferring a more upright leaf architecture (Fig. 4C). Compared to *TaBRI1-NS*, TGW was reduced by 15.9% in *tabri1-a.3bd* and 10.6% in *tabri1-bd* (not noted in GH trials) but was not significantly different in other mutant lines (Fig. 4D). The seed area was unaffected in the *bri1* mutants, except for in the *tabri1-a.3bd* mutant, where seeds were 7% smaller than *TaBRI1-NS* (Fig. 4E).

Taken together, our results show that the *tabri1* mutant phenotypes were largely consistent between GH and field conditions. The *tabri1-a.1b* and *tabri1-a.1d* double mutants exhibited increased leaf erectness with no alteration in plant height, spike, and grain characteristics.

### *tabri1* mutants display reduced sensitivity to external BR application

**Fig. 5:**
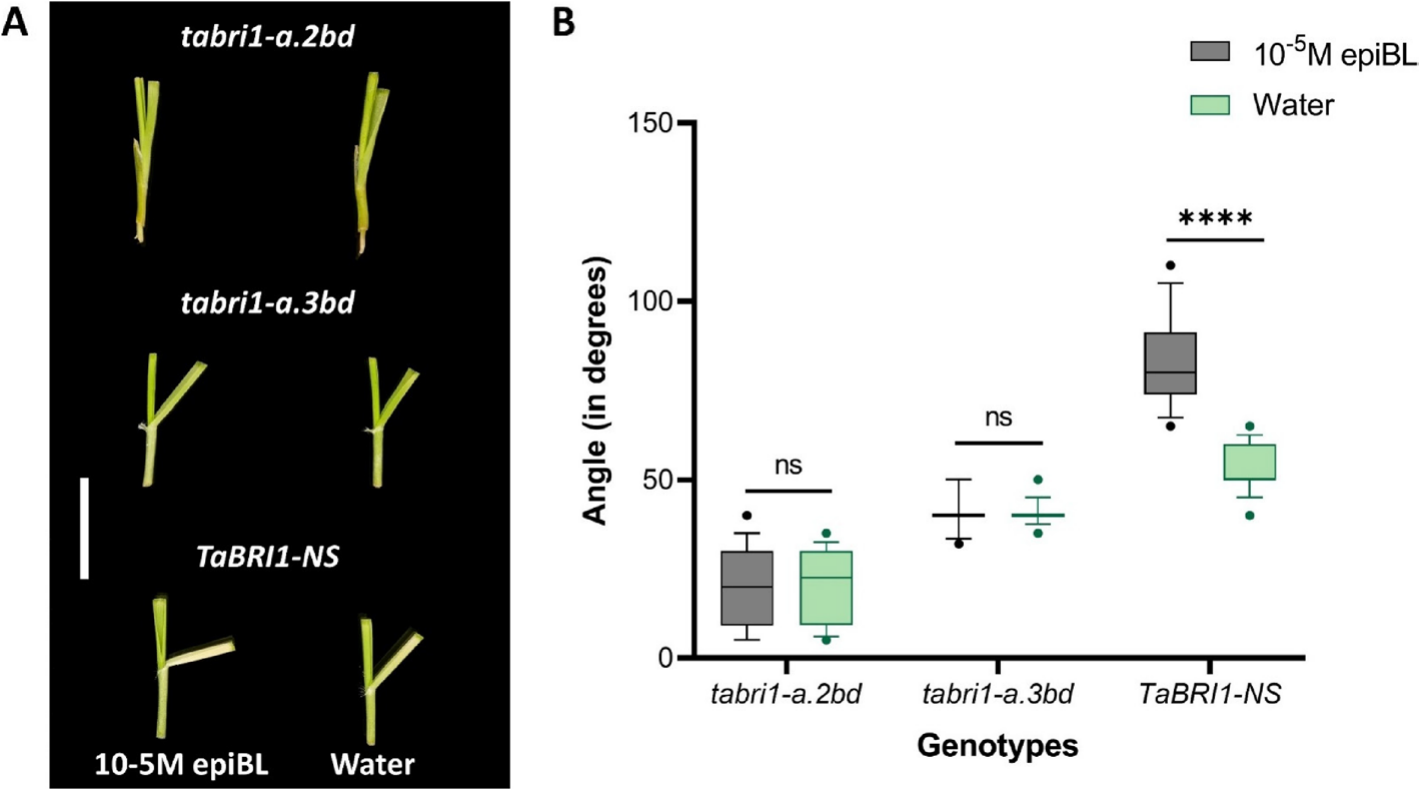
Response of *tabri1* mutants and the control line to external BR application. **A** Lamina joint inclination assays in *TaBRI1-NS*, *tabri1-a.2bd* and *tabri1-a.3bd* seedlings following treatment with 10^−5^M *epi*BL or water. Bar length is 2 cm. **B** Leaf angles (*n* = 14) after treatment with 10^−5^M *epi*BL or water. Statistical significance is denoted by adjusted *P* values: ****P < 0.0001 (obtained by pairwise Student’s t-test)

To determine whether the semi-dwarf phenotype of the triple *tabri1* mutants is caused by reduced sensitivity to endogenous BRs, we tested the effect of applied *epi*BL on leaf angle in seedlings using lamina joint inclination assays [36]. Leaf angle was significantly increased in the control *TaBRI1-NS* by 59% following *epi*BL treatment compared to the water control (Fig. 5A and B). By contrast, *epi*BL treatment did not affect leaf angle in either *tabri1-a.2bd* or *tabri1-a.3bd* triple mutant, consistent with reduced BR sensitivity.

### *tabri1* mutants accumulate intermediates and bioactive products of BR biosynthesis pathway

**Fig. 6:**
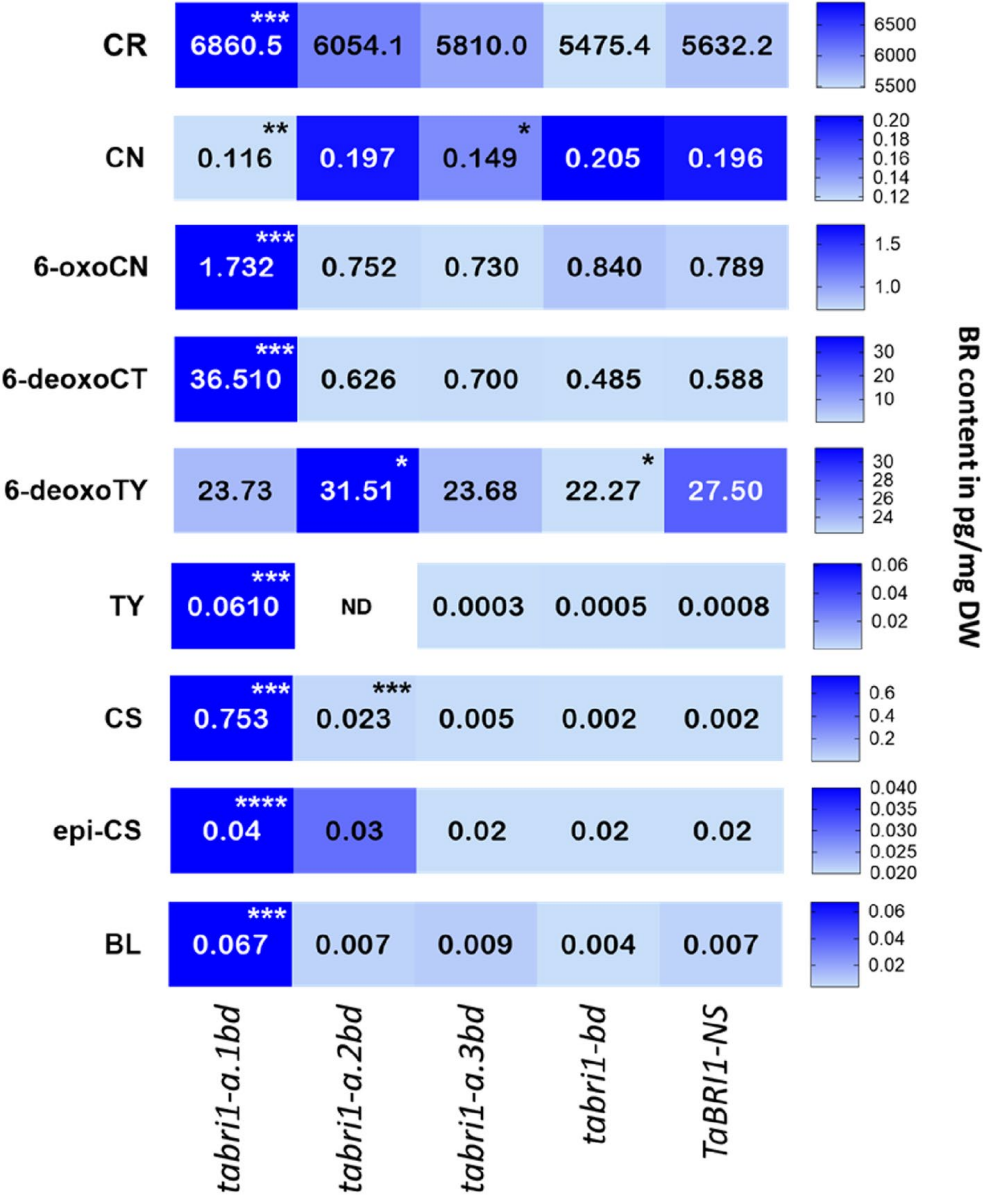
BR concentrations in *tabri1* mutants and *TaBRI1-NS.* The concentrations of BRs (pg/mg DW) namely CR (campesterol), CN (campestanol), 6-oxoCN, 6-deoxoCT (6-deoxocathasterone), 6-deoxoTY, TY (typhasterol), CS (castasterone), BL (brassinolide), and epiCS were determined in *tabri1* mutant (*tabri1-a.1bd*, *tabri1-a.2bd*, *tabri1-a.3bd, tabri1-bd*) and *TaBRI1-NS* seedlings. Mean values from five biological replicates are recorded. Statistically significant differences from the *TaBRI1-NS* control are denoted by * 0.05 > *P* > 0.01, ** 0.01 > *P* > 0.001, *** 0.001 > *P* > 0.0001, **** *P* < 0.0001 (obtained by pairwise Student’s t-test). ND = not detected

We detected nine BRs and precursors in the *tabri1* mutants and *TaBRI1-NS* (Fig. 6, Additional file 1: Table S1). The levels of CS, a bioactive BR, were increased by 376- and 12-fold in the triple mutants *tabri1-a.1bd* and *tabri1-a.2bd*, respectively, compared to *TaBRI1-NS* but its increase was not significant in *tabri1-a.3bd* (Fig. 6, Additional file 1: Table S1). We observed a 9.6-fold accumulation in BL content in the most severe triple mutant *tabri1-a.1bd* compared to *TaBRI1-NS* with no significant differences for the other *bri1* mutants. Additionally, we observed significant accumulation of TY and 6-oxoCN, which are intermediates in the early C-6 oxidation pathway, and of 6-deoxoCT, an intermediate in the late C-6 oxidation pathway, in *tabri1-a1.bd* compared to *TaBRI1-NS*.

### BR biosynthesis and signalling genes are differentially expressed in the *tabri1* mutants

To determine the effect of reduced TaBRI1 function on gene expression we sequenced the transcriptomes of *tabri1* mutant and control seedlings. The transcriptome was radically altered in the *tabri1-a.1bd* mutant which had 35,504 differentially expressed genes (DEGs) compared to *TaBRI1-NS* (Additional file 1: Figure S4C, Additional File S3). Transcriptomic differences in the *tabri1-a.1bd* mutant were also evident in the principal component analysis, which grouped the mutant separately from other genotypes in the experiment (Additional file 1: Figure S4).

By contrast, there were just 139 DEGs between the double mutant *tabri1-bd* and *TaBRI1-NS* consistent with the functional redundancy in *BRI1* genes in wheat seedling tissues (Additional file 1: Figure S4, Additional File S3). The milder *tabri1-a.3bd* mutant exhibited 2,867 DEGs, consistent with the partial disruption of BR signalling in this mutant (Additional file 1: Figure S4, Additional File S3).

**Fig. 7:**
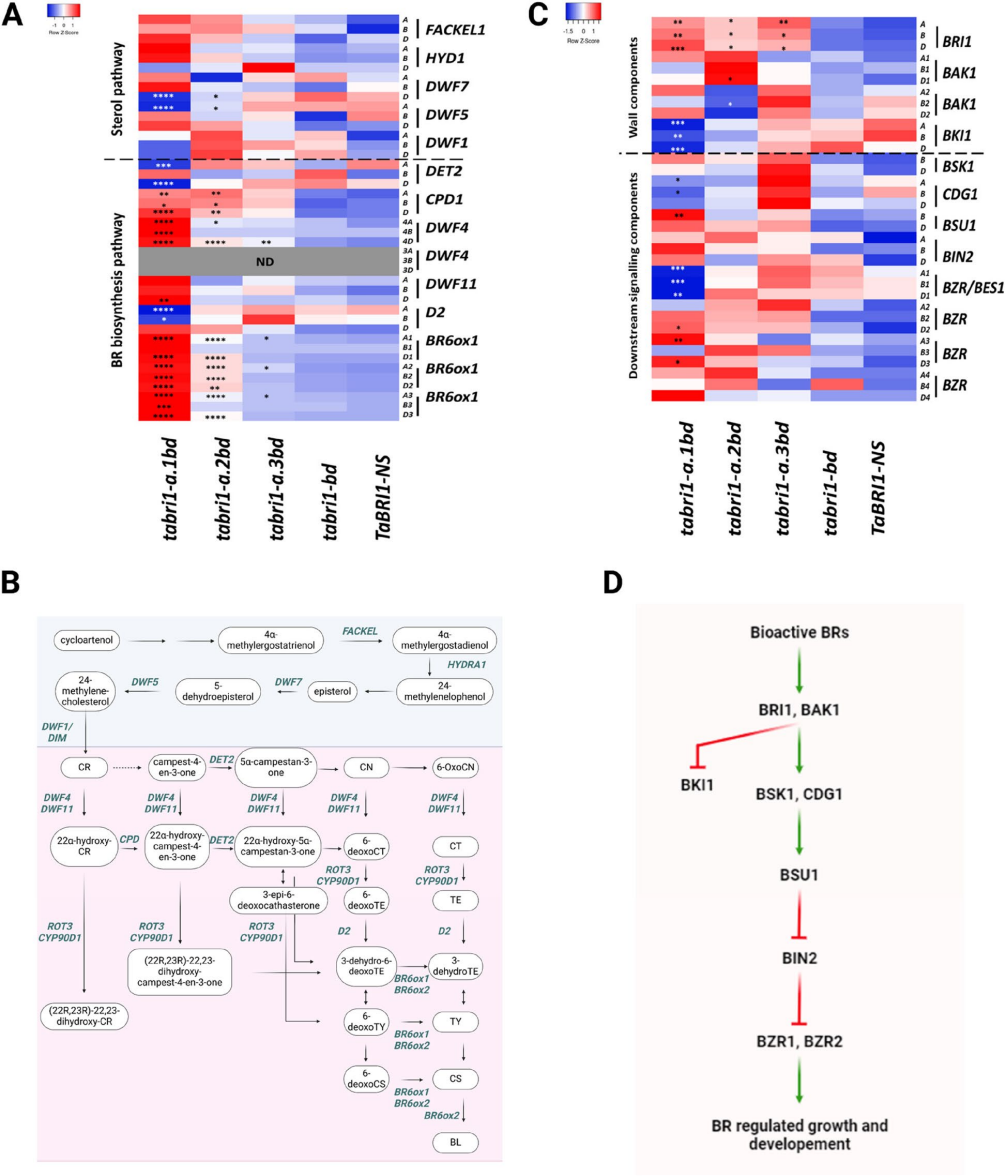
Transcript levels of BR biosynthesis and BR signalling genes in *tabri1* mutants and *TaBRI1-NS*. A Heatmap of relative expression levels of genes in the sterol and BR-biosynthesis pathways in the *tabri1-a.1bd*, *tabri1-a.2bd*, *tabri1-a.3bd*, *tabri1-bd* mutants and *TaBRI1-NS*. **B** The sterol pathway (in blue background) and BR-specific biosynthesis pathways (in pink background) leading to the bioactive BRs CS and BL. The names of the enzymes that catalyse each step is shown above the arrows. TE teasterone, *DET2 DE-ETIOLATED2*. **C** Heatmap indicating expression levels (TPM) of genes in the BR-signalling pathway in the *tabri1-a.1bd*, *tabri1-a.2bd*, *tabri1-a.3bd*, and *tabri1-bd* mutants and control *TaBRI1-NS.* Statistically significant differences from the *TaBRI1-NS* control are denoted by * 0.05 > *P-adj* > 0.01, ** 0.01 > *P-adj* > 0.001, *** 0.001 > *P-adj* > 0.0001, **** *P-adj* < 0.0001. ND = not detected. **D** BR signalling pathway in plants. BSK1 (Brassinosteroid-Signaling Kinase1), CDG1 (CONSTITUTIVE DIFFERENTIAL GROWTH1), BSU1 (BRI1 SUPPRESSOR 1), *BIN2* (*BRASSINOSTEROID-INSENSITIVE2*). The pathways were produced using Biorender.com

We hypothesised that genes differentially expressed in both *tabri1-a.1bd* and *tabri1-a.2bd* mutants which share a dwarfed phenotype might represent a core set of BR-regulated genes in wheat. In total, 4,654 genes were differentially expressed in both mutants which were significantly enriched for functional terms relating to carbohydrate metabolism (Additional file File S4). The BR biosynthesis genes *DWF4*, *CPD*, and *BR6oxs* were significantly up-regulated in both dwarfed mutant lines but not in the milder *tabri1-a.3bd* mutant, consistent with changes in bioactive BR levels (Fig. 7A, File S5). By contrast, transcript levels of the BR signalling genes *TaBZR1*, *TaBKI1* and *TaCDG1* were significantly reduced only in the most severe *tabri1-a.1bd* mutant (Fig. 7C, Additional File S5).

## Discussion

### Targeting reduced BR activity for crop improvement

Reducing BR signalling can lead to shorter plants with a more erect leaf architecture, potentially improving crop yields in certain environments. However, since BRs regulate a wide range of growth and developmental processes, completely abolishing BR signalling results in extreme phenotypes [15, 39]. Consistent with findings in Arabidopsis and rice, we observed that a complete knockout of the BR receptor BRI1 in wheat severely disrupts growth and development, producing an extreme phenotype unsuitable for wheat breeding (Fig. 1C).

Successful applications of reduced BR signalling in agriculture have been achieved through genetic changes that confer targeted or partial reductions in BR activity. For example, in maize, a loss-of-function allele of the BR biosynthesis gene *DWF4* confers a more erect leaf angle in the upper canopy without affecting lower leaves or overall height [40]. In rice, enhanced panicle branching is caused by a structural rearrangement resulting in the upregulation of *BRASSINOSTEROID-DEFICIENT DWARF3*, which encodes a BR catabolic enzyme, specifically in secondary branch meristems [41].

The functional redundancy in the polyploid wheat genome might be exploited to moderately reduce BR activity. A recent study demonstrated that loss of a haploblock including *ZnF-B*, which encodes a RING-type E3 ligase, stabilized the negative BR regulator TaBKI1. This resulted in a compact and upright growth habit and improved grain yields in the absence of *Rht1* dwarfing genes [42]. Conversely, CRISPR/Cas9-mediated knockout of all copies of *ZnF-B* resulted in reduced grain size, highlighting the importance of balancing BR levels [42].

In our study, mutations in one or two of the *BRI1* homoeologues conferred increased leaf erectness without affecting plant height or grain size (Fig. 3). This contrasts with the report by Lyu et al. [43], in which all combinations of double *bri1* mutants showed reduced height and shorter grain than the cv. Fielder wild type. This difference may be due to their genetic backgrounds: while Fielder contains the *RHT-B1b* semi-dwarfing allele, Cadenza contains homoeologous wild-type *RHT-1a* alleles. It will be important to evaluate the effects of the *bri1* alleles in field trials with varying planting densities to determine their potential breeding value. Detailed expression profiling of *BRI1* homoeologues across different plant tissues could reveal tissue-specific expression patterns that could help in selecting optimal mutant combinations to target reductions in FLA. While our RNA-seq data from seedlings show highest expression of *BRI1-D1* (Supplementary File 1, Figure S6A), the comprehensive survey available for cv. Azhurnaya (https://www.wheat-expression.com) shows only small differences in *BRI1* homoeologue expression (Supplementary File 1, Figure S6B). It may require analysis with higher spatial resolution to determine the relevance of differences in homoeologue expression patterns.

Gene redundancy may explain why *BRI1* alleles have never been selected in wheat breeding programmes, in contrast to the high frequency of *uzu* alleles in diploid barley germplasm [16].

To overcome this functional redundancy and identify beneficial alleles, we mutagenized the double *bri1-bd* mutant and selected potential *BRI1-A* mutants on the basis of phenotype. We identified two alleles (*tabri1-a.2* and *tabri1-a.3*) with amino acid substitutions which conferred reduced height and BR insensitivity (Fig. 3). In both cases, the amino acid substitutions, P671S in *tabri1-a.2bd* and G1008E in *tabri1-a.3bd*, are predicted on the basis of SIFT scores to affect protein function (Table 1). The *tabri1-a.2bd* mutation is in the final LRR domain and is predicted to disrupt BR signalling by interfering with BR binding into the protein superhelix [44, 45]. The Arabidopsis mutant *bri1-711* with a G746S substitution in the last LRR domain demonstrated a mild reduction in rosette width and height [46]. The P to S substitution in *tabri1-a.2bd* would be expected to result in a major structural change that would explain the severe phenotype. The mutation in *tabri1-a.3bd* is in the IX region of the Ser/Thr-kinase domain. In rice, the *d61-1* mutant with an amino acid substitution in the IX subdomain exhibits dwarfism, erect leaves, and reduced grain number per panicle [16, 19]. Mutations in the IX subdomain would affect BR signalling due to their proximity to the activation loop in the BRI1 kinase domain [47]. Autophosphorylation of the activation loop in BRI1 in association with the kinase domain of BAK1 stabilizes the conformation leading to the activation of BR signalling [48–50].

Although these alleles also conferred pleiotropic traits such as reduced grain size and spike length—likely precluding their use in agriculture—our approach demonstrates the potential of targeted forward genetic screens to expand genetic diversity in the BR pathway for wheat improvement. Screening a larger mutant population or using targeted genome editing strategies to introduce high value *uzu* alleles into wheat could further extend this variation and potentially identify *BRI1* alleles that improve architecture without detrimental effects.

### BR-insensitive *tabri1* mutants have higher BR levels

Nine BRs were detected in wheat seedlings, including the bioactive forms BL and CS (Fig. 6; Additional file1: Table S1). While BL has been previously detected in wheat [51], its presence in other cereals has not been reported [19, 37, 52]. Notably, intermediates in the late C-6 oxidation pathway were more abundant than those in the early C-6 oxidation pathway, indicating that both pathways are active in wheat but the late C-6 oxidation pathway predominates.

The *tabri1-a.1bd* triple mutant, exhibiting the most severe developmental defects, accumulated high levels of both bioactive BRs and their precursors (Fig. 6; Additional file 1: Table S1). This accumulation was associated with strong upregulation of *DWF4*, which encodes a 22α-hydroxylase involved in BR biosynthesis [53], *BR6OX1* paralogues and *CPD*, which encodes a C-3 oxidase [54] (Fig. 7). Similar upregulation of these genes has been observed in Arabidopsis in response to BR depletion, with *DWF4* being the most strongly and rapidly affected [8]. As a major rate-limiting enzyme in BR biosynthesis, *DWF4* is highly regulated by feedback mechanisms and other factors [55, 56]. Upregulation of *DWF4* can account for the accumulation of intermediates early in the BR biosynthetic pathway. In combination with increased expression of *BR6OX1*, which functions later in the pathway, these changes account for the large increase in bioactive BRs in the *tabri1-a.1bd* mutant. The changes in expression of BR metabolism and signalling genes were much more pronounced in the severe *tabri1-a.1bd* mutant line than in the other mutants, as reflected by the total number of DEGs: 35,504 in *tabri1-a.1bd*, compared to 2,867 in *tabri1-a.3bd* and 139 in *tabri1-bd*, relative to the null segregant. Therefore, changes in expression of BR-regulated genes, particularly those involved in feedback regulation of BR biosynthesis, require a severe reduction in BR signalling in wheat, whereas phenotypic changes, including a reduction in leaf angle, are apparent already at more modest changes in BR signalling.

## Conclusion

We have identified novel loss-of-function and reduced-function alleles of the wheat homoeologous *BRI1* genes. Plants carrying just one functional *BRI1* homoeologue exhibit a more erect leaf architecture without any pleiotropic effects on plant height or grain development. These alleles are potential candidates for yield improvement in high-density sowing regimes. By contrast, plants with combinations of loss-of-function alleles and amino acid substitutions in conserved *BRI1* domains were semi-dwarfed with more erect leaves, but negative effects on grain size and reproductive development likely preclude their use in wheat breeding. Screening for natural variation in *BRI1* genes within wheat germplasm collections or inducing specific alleles through CRISPR-Cas mutagenesis to produce different homoeologous mutant combinations, informed by our characterisation of these genes, may be promising approaches to identify beneficial alleles for wheat improvement.

## Supplementary Information

Additional file 1: Figure S1. Gross Morphology of *tabri-a.2bd* mutant. Figure S2. Phenotypic data collected on *tabri1* mutants during GH2022. Figure S3. FLA relationship between reproductive growth stages. Figure S4. RNA-seq analysis. Figure S5. Scheme used for sequencing *TaBRI1A* gene. Figure S6. Expression of *BRI1* genes in wheat. Table S1. BR levels (pg/mg DW) in *tabri1* mutants and controls. Table S2. Homeologue-specific primers designed to amplify fragments around the deleterious mutations in *TaBRI1 *genes. Table S3. Primers used for sequencing *TaBRI1A* gene. Table S4. KASP primers designed to differentiate the mutant and wild-type alleles in segregating *TaBRI1* populations.

Additional file 2. GH conditions (temperature, radiation, humidity) during 2021 and 2022.

Additional file 3. DEseq2 in *tabri1* mutants compared to *TaBRI1-NS* with *P-adj value* <0.05 and log2fold change.

Additional file 4. GO terms for 1312 and 4653 core DEGs.

Additional file 5. Log_2_-fold change and *P-adj* values for BR pathway genes in *tabri1* mutants compared to *TaBRI1-N*S.

## Data Availability

RNA-seq reads and expression data for all libraries are available for download from the National Center for Biotechnology Information’s Gene Expression Omnibus under accession number GSE274403 (https://www.ncbi.nlm.nih.gov/geo/query/acc.cgi?acc=GSE274403). All other data generated in this study are provided in the manuscript and supporting tables. Biological materials are available upon request from Stephen Pearse.
