# Supplementary material for: Induced variation in BRASSINOSTEROID INSENSITIVE 1 (BRI1) confers a compact wheat architecture
Source: BMC Plant Biol. 2025 May 26;25:700. doi: 10.1186/s12870-025-06762-w (PMC12105372; doi:10.1186/s12870-025-06762-w)
Supplement: Supplementary file 1 — Additional file 1: Figure S1. Gross Morphology of tabri-a.2bd mutant. Figure S2. Phenotypic data collected on tabri1 mutants during GH2022. Figure S3. FLA relationship between reproductive growth stages. Figure S4. RNA-seq analysis. Figure S5. Scheme used for sequencing TaBRI1A gene. Figure S6. Expression of BRI1 genes in wheat. Table S1. BR levels (pg/mg DW) in tabri1 mutants and controls. Table S2. Homeologue-specific primers designed to amplify fragments around the deleterious mutations in TaBRI1 genes. Table S3. Primers used for sequencing TaBRI1A gene. Table S4. KASP primers designed to differentiate the mutant and wild-type alleles in segregating TaBRI1 populations. [file 12870_2025_6762_MOESM1_ESM.docx]

**Induced variation in *BRASSINOSTEROID INSENSITIVE 1* (*BRI1*) confers a compact wheat architecture**

**Additional file 1**

**Figure S1.** Gross Morphology of *tabri-a.2bd* mutant.

**Figure S2.** Phenotypic data collected on *tabri1* mutants during GH2022.

**Figure S3.** FLA relationship between reproductive growth stages.

**Figure S4.** RNA-seq analysis.

**Figure S5.** Scheme used for sequencing *TaBRI1A* gene.

**Figure S6**. Expression of BRI1 genes in wheat.

**Table S1**. BR levels in *tabri1* mutants and controls.

**Table S2**. Homeologue-specific primers designed to amplify fragments around the deleterious mutations in *TaBRI1* genes.

**Table S3**. Primers used for sequencing *TaBRI1A* gene.

**Table S4**. KASP primers designed to differentiate the mutant and wild-type alleles in segregating TaBRI1 populations.

**Figure S1.** **Gross Morphology of *tabri-a.2bd* mutant** (A) Closeup photo of *tabri1-a.2bd* mutant grown in the glasshouse and photographed at GS83 during GH2021. (B) Curled and wrinkled flag-leaf of *tabri1-a.2bd* mutant.


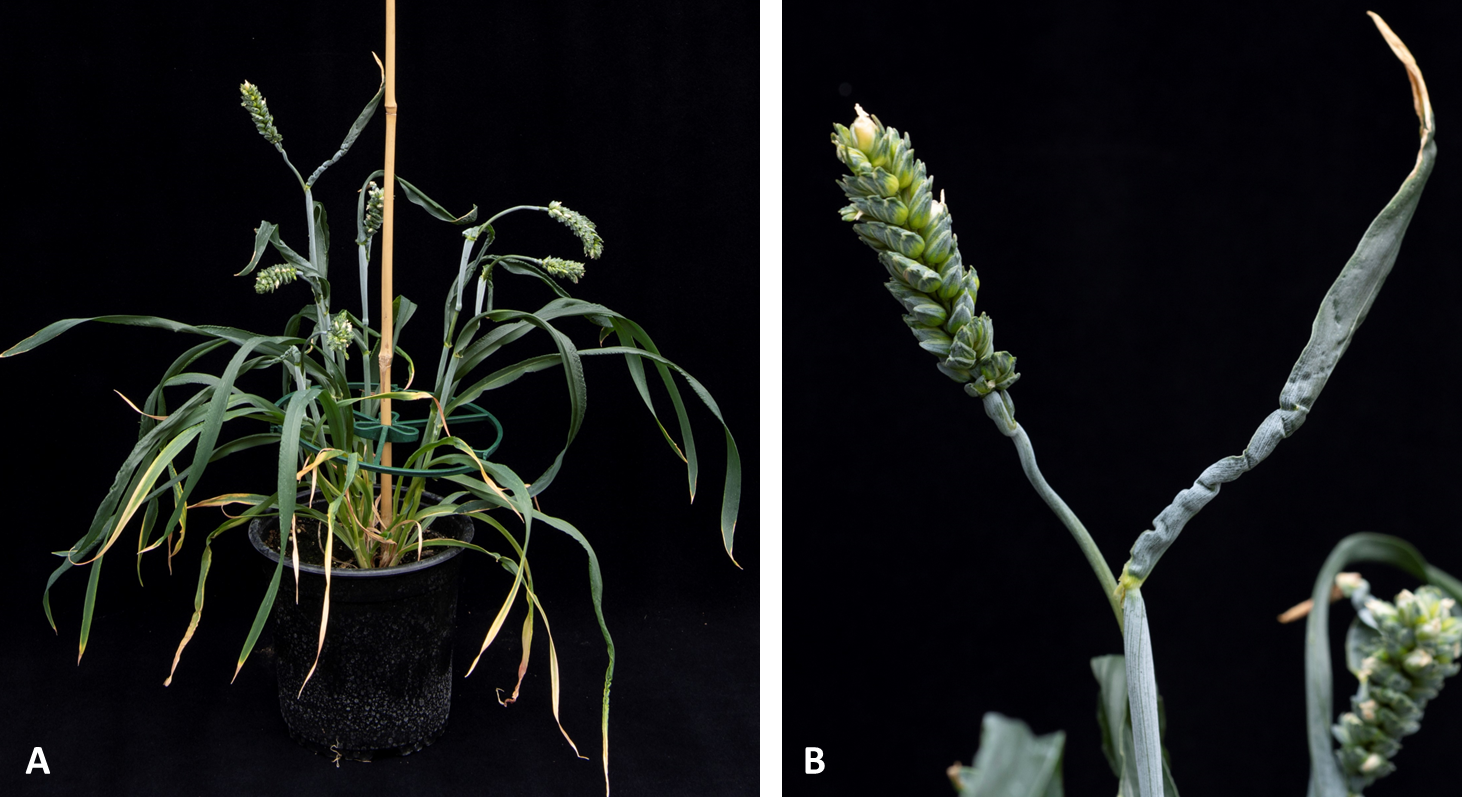


**Figure S2.** **Phenotypic data collected on *tabri1* mutants during GH2022.** **(A)** Final plant heights at maturity (n=6). **(B)** Spike length recorded at maturity (n=6). **(C)** Number of spikelets/spike recorded on mature spikes (n=6). **(D)** FLAs at anthesis (n=6). **(E)** 1000 grain weight (TGW) recorded on mature grains (n=6). **(F)** Seed area obtained using the Marvin seed analyser on mature grains (n=6). *P*-values for differences between mutants and *TaBRI1-NS* were obtained using Fisher’s unprotected LSD test are also shown on the graph.


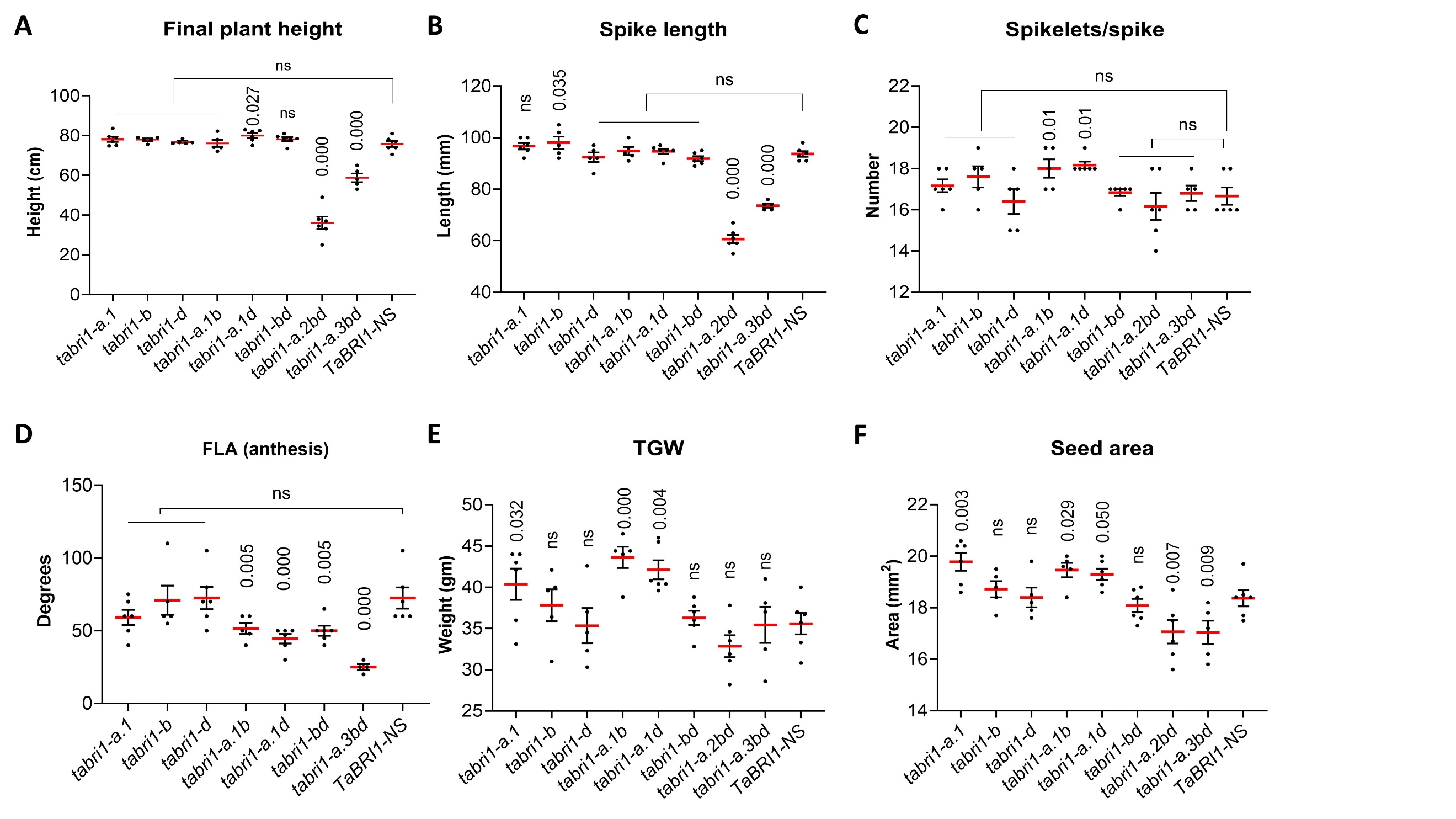


Figure S3. FLA relationship between reproductive growth stages. Relationship of Flag leaf angle between ear emergence and anthesis (A), anthesis and water endosperm (B), watery endosperm and soft dough (C) and soft dough and ripening (D) growth stages. Very strong 1:1 relationship was observed in flag leaf angle between subsequent growth stages (*p* < 0.001).

**A**
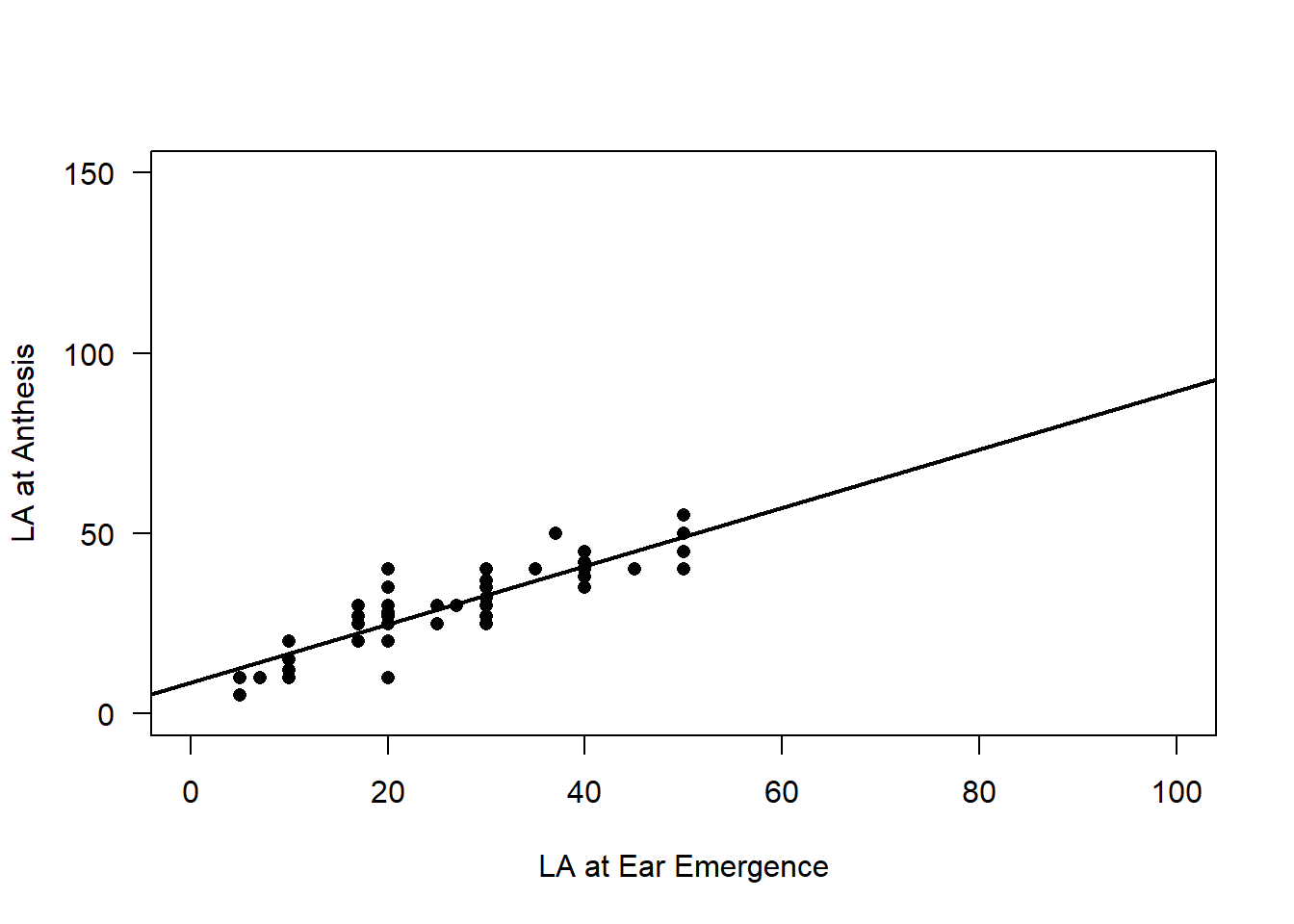
 **B**
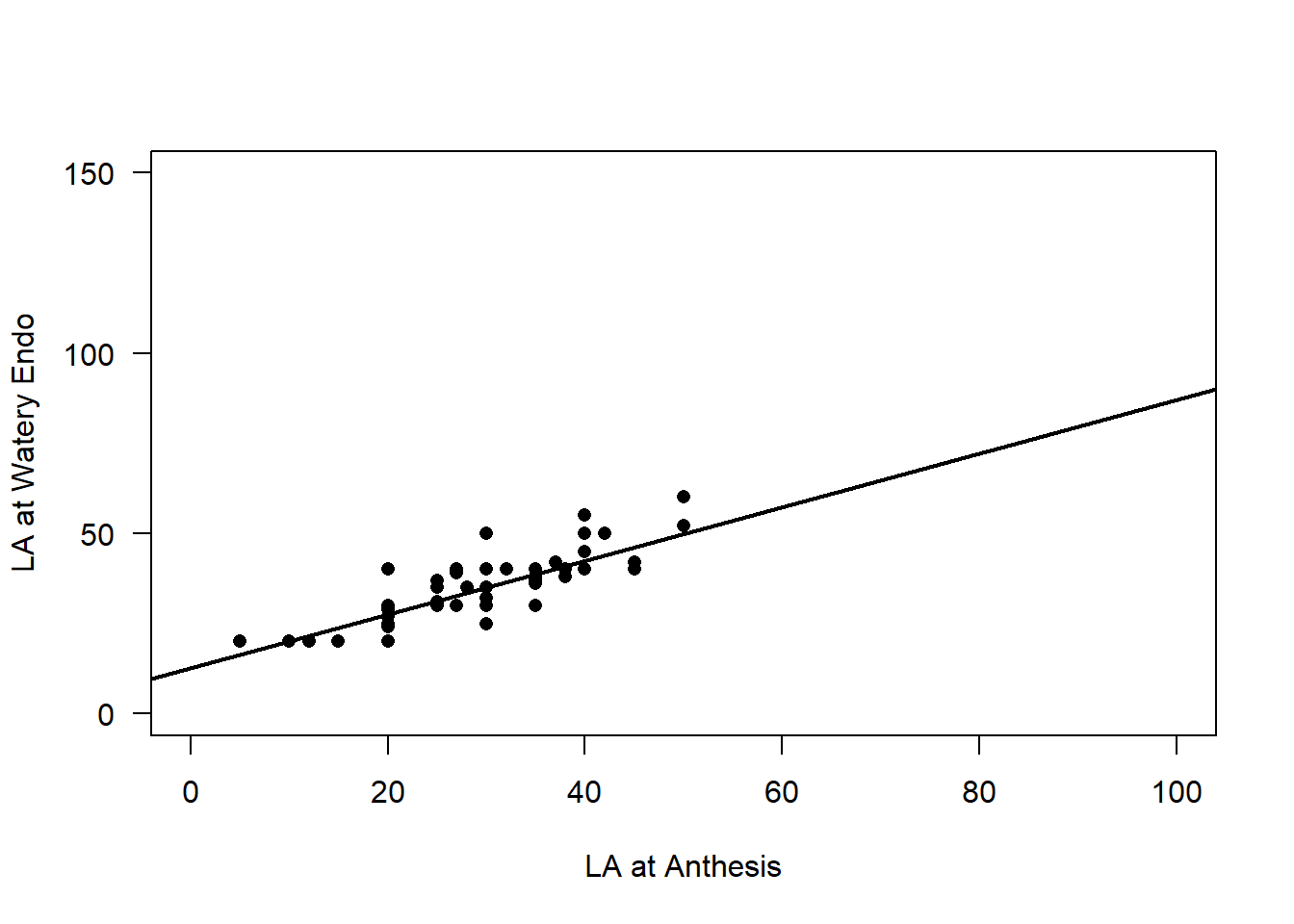


**C**
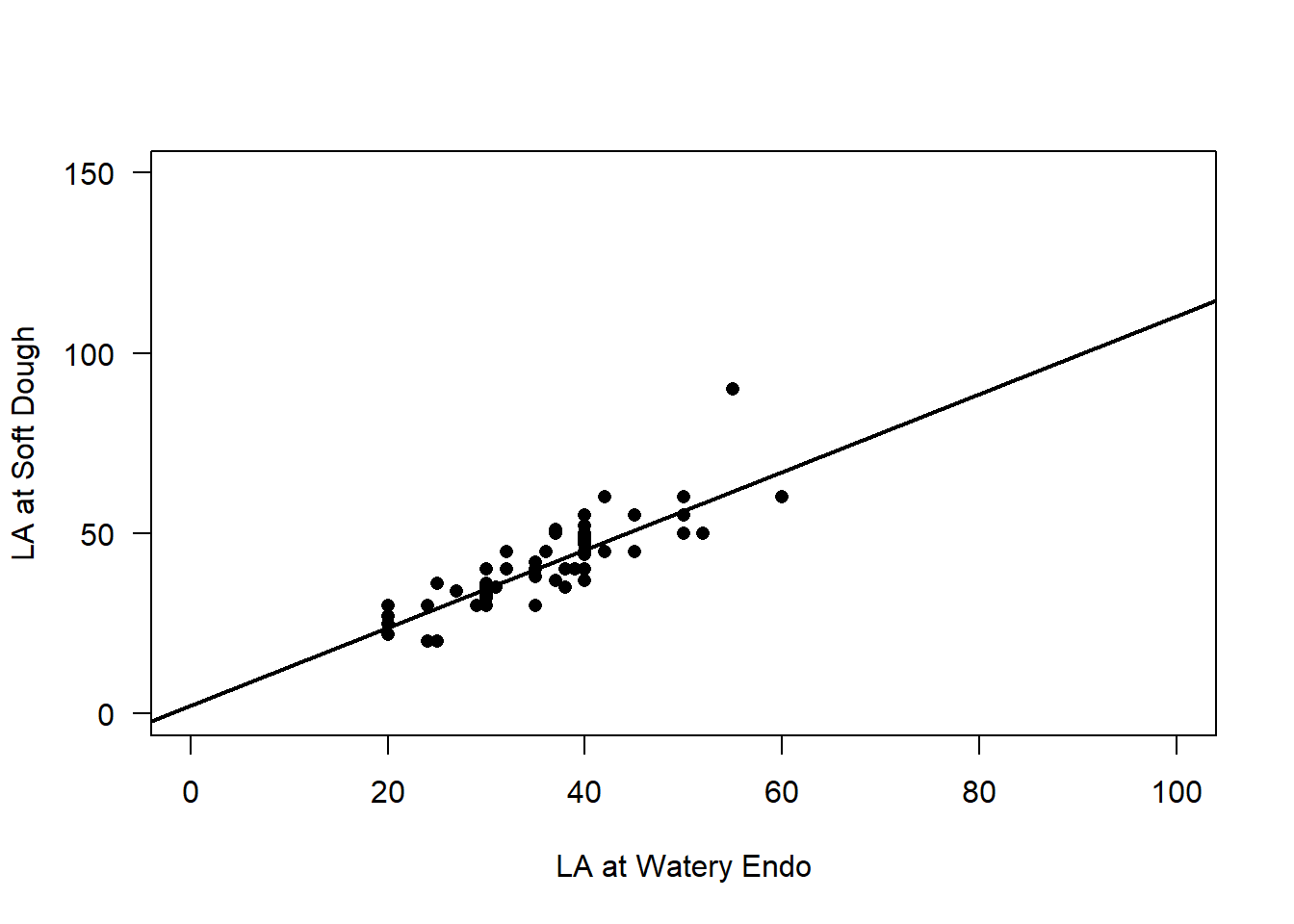
 **D**
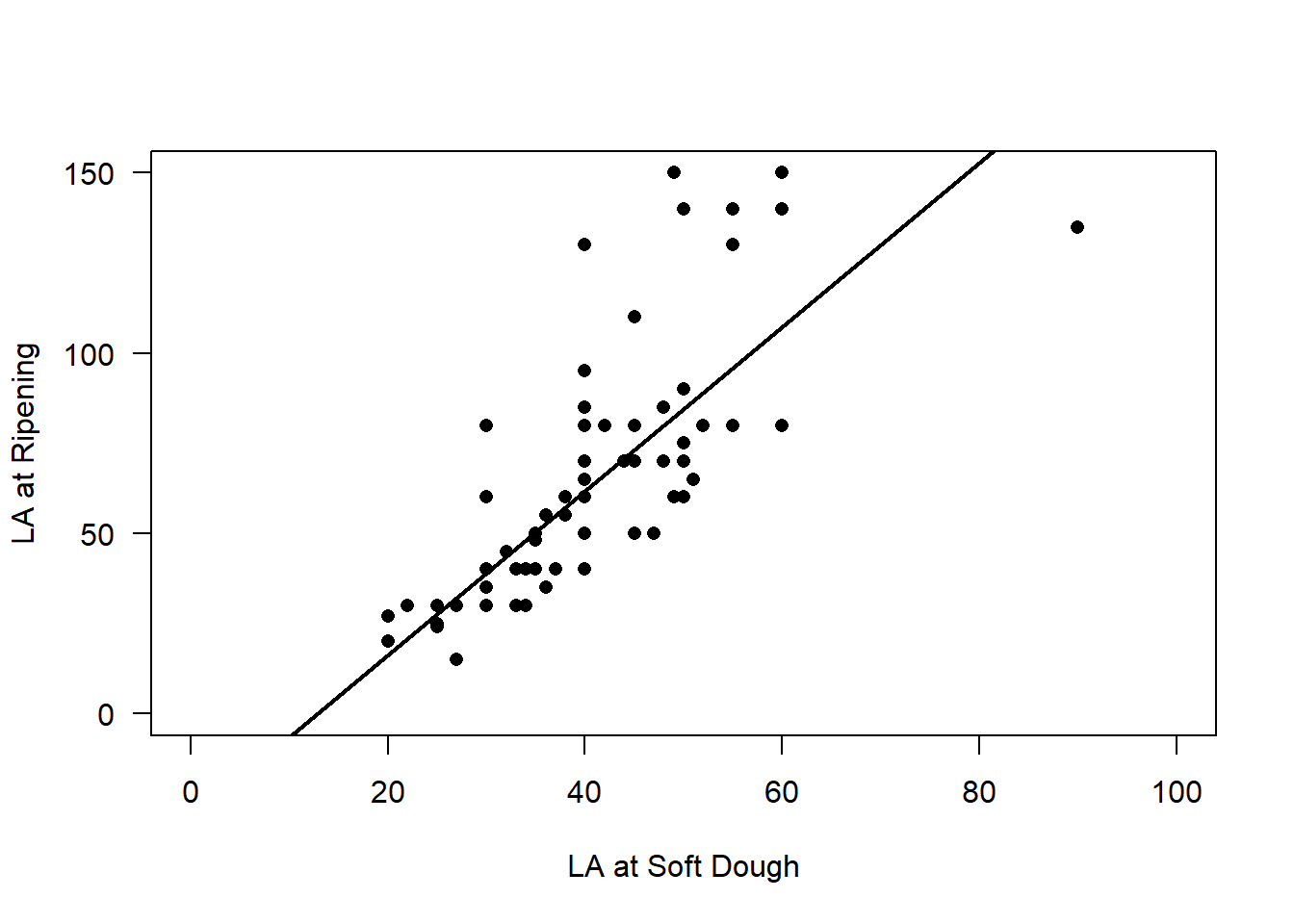


**Figure S4. RNA-seq analysis.** **(A)** Venny2.1 (<https://csbg.cnb.csic.es/BioinfoGP/venny.html>) was used to produce Venn diagrams for the genes co-expressed in the *tabri1-a.1bd, tabri1-a.2bd* and *tabri1-a.3bd* mutants compared to *TaBRI1-NS*. **(B)** PCA plot was generated using Bioconductor package pheatmap version 1.0.12 to show the overall variation in gene expression in *tabri1-a.1bd, tabri1-a.2bd, tabri1-a.3bd*, *tabri1-bd* mutants compared to *TaBRI1-NS* and Cadenza. **(C)** Stacked bar graph was produced in Microsoft excel 365 to show number of differentially expressed genes (with *p*-value >0.05) in *tabri1-a.1bd, tabri1-a.2bd, tabri1-a.3bd* and *tabri1-bd* mutants compared to *TaBRI1-NS*.


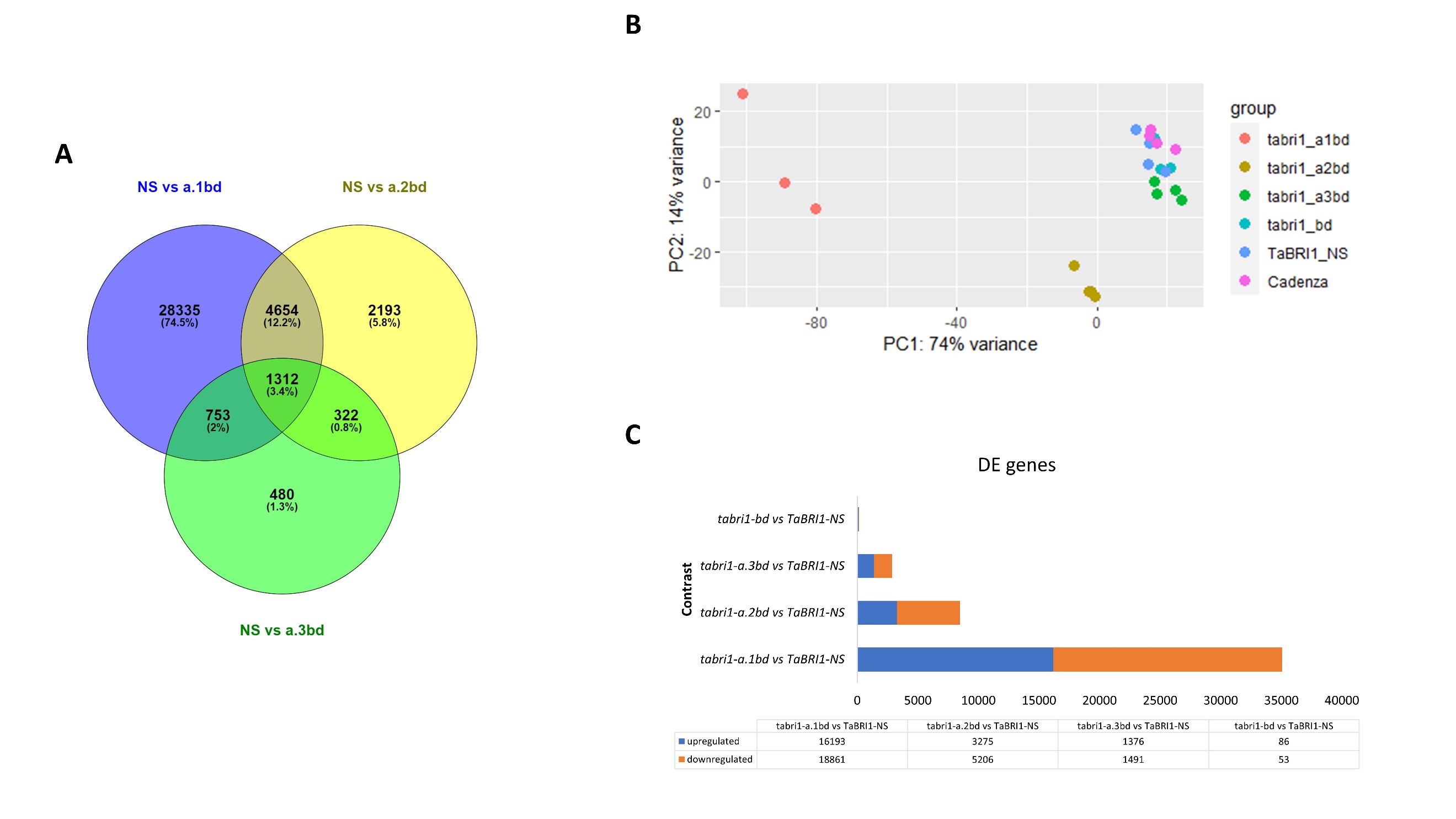


**Figure S5.** **Scheme used for sequencing *TaBRI1A* gene.** Primer pairs F1-R1, F2-R2, F3-R3 and F4-R4 were used to amplify fragments in *TaBRI1A* gene from shortlisted M3 mutant lines. Sanger sequencing of the fragments was performed using F1, R1 (and IF1, IR1), F2, R2 (and IF2, IR2), F3, R3 (and IF3, IR3), F4, R4 (and IF4, IR4). The sequence of primers and conditions for PCR reactions used are mentioned in Table S3.


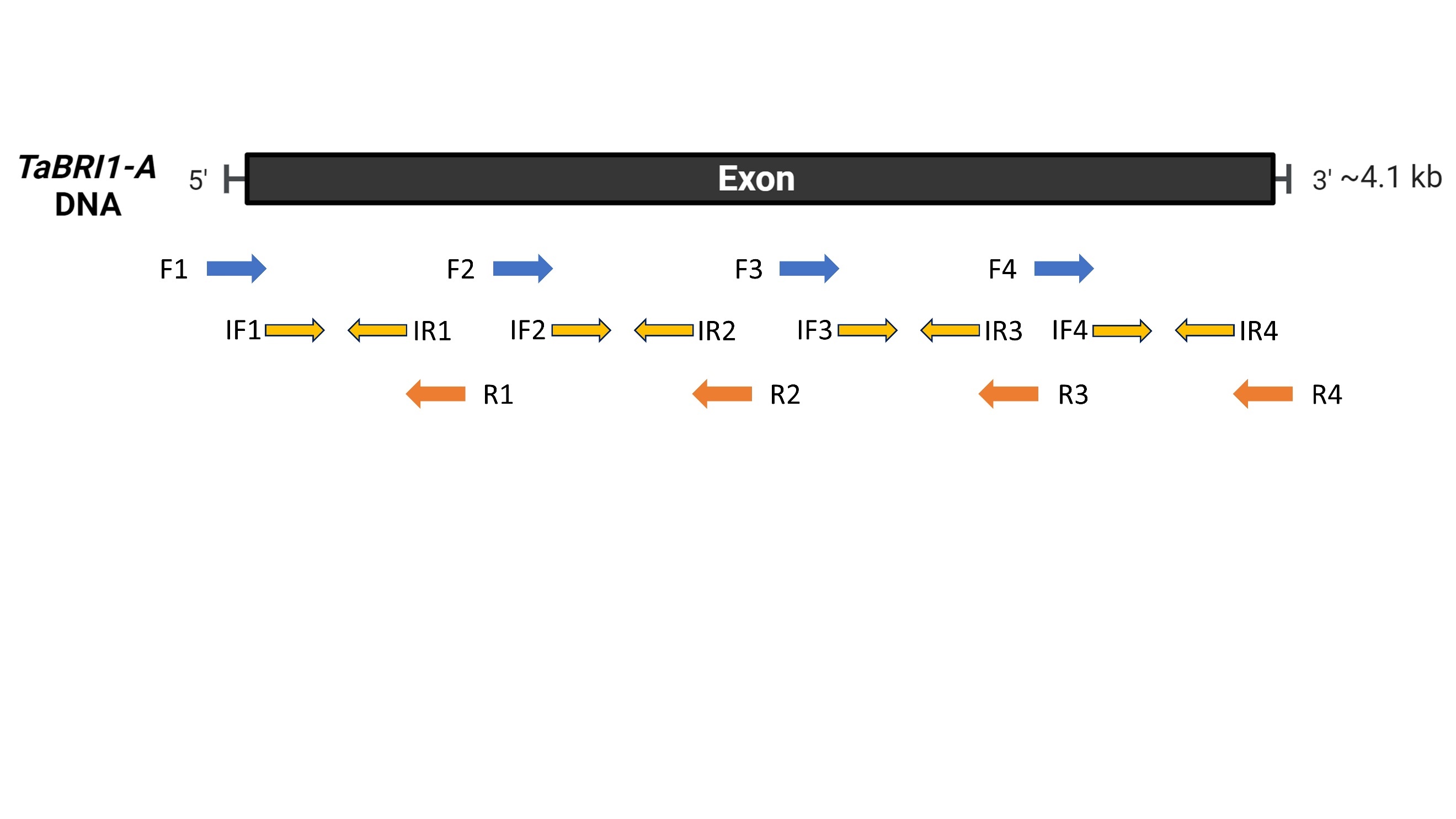


**Figure S6. Expression of *BRI1* genes in wheat.** (A) Shows expression of *BRI1* genes (in TPM or transcripts per million) at the seedling stage, obtained from our RNA-seq experiment in *TaBRI1-NS* and Cadenza. (B) Shows expression of *BRI1* genes (in TPM) over the developmental time course in cv ‘Azhurnaya’ available at (<https://www.wheat-expression.com>).


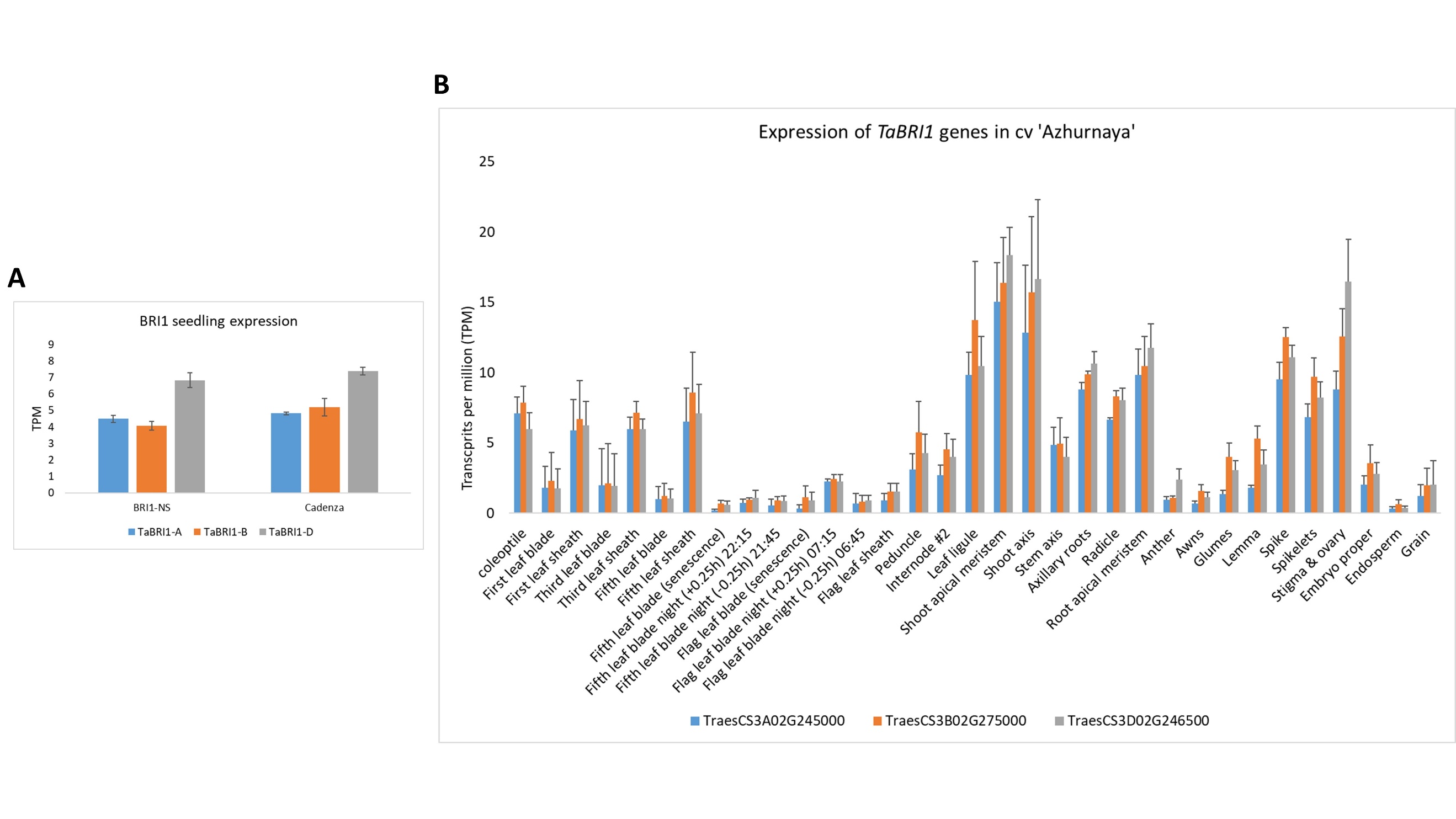


**Table S1.** **BR levels (pg/mg DW) in *tabri1* mutants and controls.** Mean values (pg/mg DW) ± SD are shown for four *tabri1* mutants along with controls i.e., *TaBRI1-NS* and Cadenza at the seedling stage. The data were analysed using one-way ANOVA which yielded *p*-values, SED, and LSD at 5% level of significance. Fisher’s unprotected LSD test was performed for multiple pair-wise comparisons. Statistically significant difference between mutants and *TaBRI1-NS* were denoted **P*< 0.05, ***P*< 0.01, ****P*< 0.001, *****P* < 0.0000 (obtained from Fisher’s LSD unprotected test).


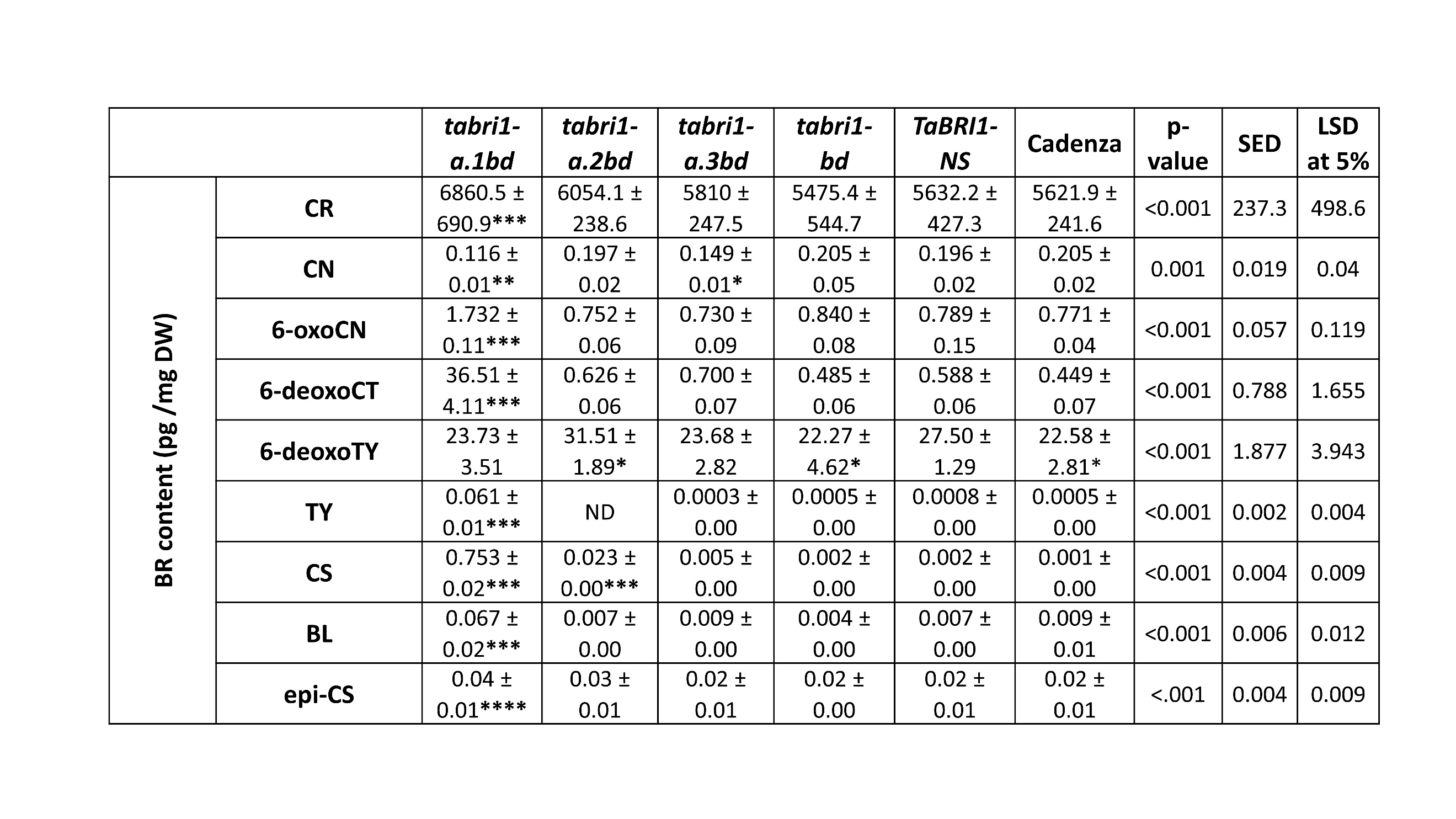


**Table S2.** **Homoeologue specific primers designed to amplify fragments around the deleterious mutations in *TaBRI1* genes.** The gene information, primer sequence, fragment length produced, and Tm (℃) used for PCR reaction is mentioned below.

**Table S3.** **Primers used for sequencing *TaBRI1A* gene.** The primer sequence, fragment length and Tm (℃) used for PCR reaction is mentioned below. Internal primers were used only for sequencing.

**Table S4.** KASP primers designed to differentiate the mutant and wild type allele in segregating *TaBRI1* populations.
